# Evolutionary Specializations of the Human Vertebral Body and Intervertebral Disc in Relation to Bipedalism

**DOI:** 10.3390/life16030466

**Published:** 2026-03-12

**Authors:** Israel Hershkovitz, Bruce Latimer, Janan Abbas, Mila Hejja, Bahaa Medlej, Hanan Rapoport, Einat Kedar, David Ezra, Ian Rybak, Tatiana Sella Tunis, Irit Zohar, Gali Dar

**Affiliations:** 1 Department of Anatomy and Anthropology, Gray Faculty of Medical and Health Sciences, Tel Aviv University, Tel Aviv 6997801, Israel; 2 The Shmunis Family Anthropology Institute, Dan David Center for Human Evolution and Biohistory Research, Tel Aviv University, Tel Aviv 6997801, Israel; 3 Department of Physical Anthropology and Orthopedic Surgery, School of Medicine, Case Western Reserve University, Cleveland, OH 44106, USA; 4 Department of Physical Therapy, Zefat Academic College, Zefat 1320611, Israel; janan1705@gmail.com; 5 Department of Anatomy, Faculty of Health and Medical Sciences, Arab American University of Palestine, Ramalla 00970, Palestine; 6 School of Nursing Sciences, Academic College of Tel Aviv-Jaffo, Jaffo 6818211, Israel; 7 Department of Orthodontics, The Maurice and Gabriela Goldschleger School of Dental Medicine, Gray Faculty of Medical and Health Sciences, Tel Aviv University, Tel Aviv 6997801, Israel; tanya.tuniss@gmail.com; 8 Department of Biology and Environment, University of Haifa, Oranim 3604301, Israel; zoharir@tauex.tau.ac.il; 9 Marine Biodiversity Center, The Steinhardt Museum of Natural History, Tel Aviv University, Tel Aviv 6997801, Israel; 10 Department of Physical Therapy, Faculty of Social Welfare & Health Sciences, University of Haifa, Mount Carmel, Haifa 3103301, Israel; gdar@univ.haifa.ac.il

**Keywords:** human evolution, bipedal walking, spine, vertebra, intervertebral disc, apophyseal ring, endplate, annulus fibrosus, upright posture, spine pathologies, trade-offs

## Abstract

It is widely accepted that modern humans display distinctive vertebral and intervertebral disc (IVD) morphologies that evolved to meet the biomechanical demands of habitual terrestrial bipedalism. This study synthesizes macro- and microstructural differences in the lumbar spine to clarify how human specializations compare with those of extant apes. The skeletal sample consisted of 240 humans, 20 chimpanzees, and 25 gorillas. The CT scan sample comprised 180 humans and eight chimpanzees. Histological analysis of the IVD was performed on 10 humans and four ape specimens. Vertebral bodies and discs were measured. Histological analyses employed hematoxylin–eosin, Von Kossa, and Van Gieson staining. Statistical analyses included ANOVA with Bonferroni-corrected *t*-tests or Welch’s ANOVA and Games–Howell post hoc tests. Regression analyses were performed using ordinary least-squares estimation, and differences between regression lines were assessed using ANCOVA. Humans and chimpanzees differed significantly in vertebral body proportions, bone volume fraction, IVD thickness, apophyseal ring thickness, annulus fibrosus lamellar organization, endplate and subchondral bone thickness, and vascularization at the bone–endplate interface. These results indicate substantial evolutionary modification of the human vertebral body and IVD, enhancing rotational mobility and resistance to axial loading, key functional requirements for maintaining upright posture and efficient bipedal locomotion.

## 1. Introduction

### 1.1. Perspectives on Human Locomotion

Human bipedality represents a highly unusual form of mammalian locomotion, one that, to current knowledge, has not been independently explored by any extant mammal outside the hominin lineage [1,2]. It causes humans to be relatively slow runners and predisposes them to numerous injuries that are uncommon in other mammals [3,4,5,6,7,8,9,10,11,12]. This locomotor specialization imposes a distinctive set of kinematic and kinetic loading scenarios on the human body [12]. Easily recognizable modifications that enable this unusual form of locomotion are evident in the human foot, ankle, knee, hip, and spine [11,13,14,15,16,17]. The transition to a vertically oriented (orthogonal) spine reoriented the gravitational vector by approximately 90 degrees relative to its original pre-orthotic axis, a shift that drove substantial adaptive changes in human vertebrae and intervertebral discs [9,13,14,18,19,20]. Many of these anatomical changes are unique to modern humans and their bipedal ancestors.

The present study examines potential macro- and microstructural adaptations in the spinal motion segment (which consists of two vertebrae and an intervertebral disc—IVD) that collectively facilitated efficient bipedal walking and running. We intentionally avoid broader debates concerning the origin of bipedalism, including its chronological emergence, adaptive significance, locomotor precursors, or the locomotor repertoires of extinct hominin taxa. Instead, our focus is on the mechanical and structural modifications that support habitual bipedality in modern humans. In the Introduction, we synthesize current knowledge regarding the mechanical strains, functional constraints, and evolutionary trade-offs associated with habitual bipedal locomotion, which form the conceptual framework for our hypotheses. The aim of the present study is to explore potential adaptations associated with bipedalism in the spinal motion segment of *Homo sapiens* through comparative analysis, a research strategy widely used in paleoanthropology to generate functional hypotheses (e.g., [21]).

### 1.2. The Human Spine vs. The Chimpanzee and Gorilla Spines

The human vertebral column differs from those of chimpanzees and gorillas in several important ways [13,14,20,21,22,23,24]. Among the defining characteristics of the human spine are: its long lumbar segment (five lumbar elements); its remarkable flexibility; its unique series of alternating sinusoidal curvatures; its large vertebral body size relative to body weight; its large IVDs; its thin annular rings; its thin cortical shells (on average 0.5 mm); and its relatively low bone volume fraction [25,26,27,28].

These features contrast markedly with those seen in gorillas and chimpanzees. In those apes, the lumbar column is foreshortened (3–4 lumbar elements); the individual vertebral elements are relatively small with consequently small IVDs; wide annular rings; an extremely stiff, inflexible, poker-like spine; and relatively (compared to humans) high bone volume fractions [9,13,14,23,25,29,30,31,32,33,34,35,36,37,38].

The stiff, largely immobile thoracolumbar spine of the ape is regarded as an adaptation to arborealism in a large-bodied primate, necessitating the reduction of bending stresses along the lower spine during bouts of bridging and climbing [13]. Alternatively, the inflexible African ape spine may be an adaptation for the peculiar locomotor form practiced by these animals, knuckle-walking. During the crutch/swing phase of knuckle-walking, a stiff vertebral column and a shortened lumbar segment would protect the thoracolumbar region from repetitive and potentially damaging flexion and extension movements [39]. No matter if the ape’s shortened and stiffened lumbar was originally an adaptation for climbing, knuckle-walking, or both, owing to their lack of thoracolumbar spinal mobility, apes are largely insulated from much of the spinal pathology that accompanies the human’s highly flexible spinal column (osteoarthritis, disc herniation, spondylolysis, spondylolisthesis, etc.) [8,18,39,40].

### 1.3. Major Evolutionary Challenges Along the Way to Acquiring Efficient Bipedal Walking

Mobility: The human spine is extremely mobile and, indeed, stands alone among mammals in its unusual flexibility. In its normal condition, it is capable of large, combined angular displacements in the sagittal (flexion and extension), coronal (lateral flexion), and transverse (torsion) planes [41]. This extreme global mobility is a consequence of the complex, highly repetitive motions required during bipedal, upright walking and running [42]. Such movements include torsional stresses on the IVDs (oppositional rotation of the thorax and lower limbs), as well as the flexion and extension movements described above [43,44,45,46].

Control and balance: In addition to the skeletal features described above, much of the control and balance of our upper bodies reside in the muscular envelope connecting our torsos to our pelvis. Contractions of these muscles (e.g., internal and external obliques, transversus abdominis, rectus abdominis, erector spinae) seek to confine the primary loading vectors within or near the disc’s nucleus pulposus (NP), thereby distributing loads and reducing potential shear and/or bending on the discs and the adjacent vertebral bodies [47]. The normative motion of the vertebral segments not only relies on the dynamic activity of the associated soft tissues but also on their ability to dissipate energy through eccentric and isometric contractions and thereby prevent injury to the adjoining elements. That is, eccentric and isometric contractions of the circum-thoracic muscular envelope are a highly effective damping mechanism that, by functioning as an energy absorber, can prevent damage to the IVDs and, by extension, the associated vertebrae [47]. When this muscular envelope is fatigued (becoming less competent due to overuse, senescence, or novel loading conditions), the increased strain can result in overloading of the IVD and potential herniation of the NP through the annulus fibrosus (AF). Wedge fractures of the associated vertebrae can also occur under these circumstances. Such wedge fractures occur at highly specific locations along the column in humans, a situation not encountered in apes. Indeed, no spontaneous, non-traumatic vertebral wedge fracture has ever been reported in an ape [25,26,48,49].

In addition, compared with African apes, humans have further invaginated their thoracic columns anteriorly into the thoracic cage (e.g., [14,30,50,51]). This anatomical adaptation more closely approximates the vertebral column to the centers of rotation and, in doing so, reduces torsional and bending stresses on the IVDs. It also increases the mechanical advantage of the erector spinae, a parasagittal, epaxial muscle group that helps balance the torso over the single supporting limb [44].

The broad transverse diameter of the human thoracic cage [51,52] increases the radial distance of the circum-thoracic abdominal musculature from the spinal axis, enlarging their moment arms and thereby enhancing the mechanical advantage of the internal and external oblique and transversus abdominis muscles for controlling transverse-plane and lateral truncal movements [53,54,55]. Age-related alterations in the mechanics of the vertebral column (e.g., osteophytic fusion of the vertebral bodies or increased kyphosis and decreased lordosis owing to senescent fractures) can perturb this important energy-dissipating system [56].

It should be noted that the typical quadrupedal spine is adapted for stereotypical flexion and extension movements along what is essentially a parasagittal plane [57]. Moreover, because their spinal columns are characterized by a single, shallow kyphotic curve, any axial loading will act to deepen (compressive) or straighten (tensile) the kyphosis and thereby constrain the directionality of the primary loads along the parasagittal plane. Thus, by having a single kyphotic curve along the thoracolumbar column, quadrupeds can direct and control loading vectors along a plane roughly coplanar with the spinal axis [57]. In this situation, the epaxial and hypaxial muscle groups can control the directionality of the loading vectors near the centroids of the IVDs and associated vertebrae, thereby reducing potentially damaging off-axis stresses [58]. These muscles can simultaneously dissipate and absorb potentially damaging loads through eccentric and isometric contractions of the ventral and dorsal muscular bundles [58,59,60,61]. Appropriate control of whole-body angular momentum about a body center-of-mass is critical for generating rotationally stable bipedal locomotion [62].

Loading: In direct contrast to the described condition for quadrupeds, bipedal walking and running subjects the vertically aligned, sinuously curved spinal columns of humans to notoriously variable loading scenarios, particularly during the single-support phase of the gait cycle [63,64]. As noted above, ground reaction forces (GRFs) at heel-strike act to twist the pelvis toward the supporting side (and the suprajacent torso in the opposite direction) and, in so doing, subject the IVDs to repetitive, alternating torsional stresses [15,43,63]. These forces are cyclically reversed during contralateral heel-strike. As mentioned above, a pelvic list toward the unsupported side during the swing phase imposes lateral bending moments on the human lower spine. It is therefore clear that humans have largely forfeited the ability of quadrupeds to constrain the directionality of forces along the vertebral axis [65]. Complex combinations of anatomical features have evolved in humans to compensate for this loss [66,67]. The load on the human spine is mainly caused by gravitational forces due to the mass of the body segment, external forces and moments induced by a physical activity, and muscle tension [68].

Rotational movements: Bipedality exposes human motion segments to large torsional (rotational) movements that do not occur in other primates. In the transverse plane, habitual striding bipedality necessitates that the upper body counter-rotate with the lower body during walking and running. That is, during locomotion, the upper body of the human biped is 180 degrees out of synch with the lower body, something easily observed in walkers and runners when the contralateral upper and lower limb segments swing forward simultaneously. During heel strike in bipedal gait, ground reaction forces (GRFs) oriented posteriorly and superiorly and applied laterally to the body’s midsagittal axis generate an external axial moment that induces transverse-plane rotation of the pelvis and trunk toward the stance limb. This externally applied moment introduces angular momentum about the vertical axis, which, if not appropriately attenuated through segmental coordination, would result in increased torsional loading of the spinal structures [69,70]. Normal human gait is therefore characterized by coordinated counter-rotation of the pelvis and thorax, accompanied by reciprocal arm swing, which primarily functions to balance the angular momentum generated by the swinging legs and reduce the muscular torques required to control trunk rotation. By limiting excessive transverse-plane trunk motion and associated muscular demands, this coordinated upper-body motion may, in turn, reduce torsional loading transmitted through the lumbar spine and IVDs during walking [71,72].

This unusual bipedal adaptation subjects the thoracolumbar IVDs to alternating torsional stresses, also not experienced by our pre-bipedal ancestors. Additional torsional stresses are encountered as a result of the sinusoidal curvatures (sagittal plane) mentioned above. These curves cause certain regions of the column to deviate farther from the vertical axis of rotation, potentially leading to a “cam-like” mechanism that can induce stress concentrations along the column.

### 1.4. Evolution of Bipedal Walking Viewed in Three Dimensions

Sagittal plane: During the transition from a pronograde, quadrupedal spine to an orthograde, bipedal spine, early adaptations primarily functioned to balance the head, arms, and torso over extended hind limbs [14]. Maintaining trunk balance over the hips and a centrally placed stance foot, particularly during single-limb support, posed biomechanical challenges not encountered by pre-bipedal ancestors. In humans, a distinctive series of alternating sagittal curvatures (thoracic kyphosis and lumbar lordosis) permits upper-body balance over the lower limbs. However, these curvatures also generate regionally concentrated stresses on the IVDs and adjacent vertebrae, particularly at the apex of the thoracic kyphosis (T7–T9), the thoracolumbar junction (T11–L1), and the lumbosacral region (L4–S1). Extant apes lack a lumbar lordosis and, when attempting bipedalism, rely on a bent-hip, bent-knee gait to maintain balance. It is unlikely that early hominins regularly employed such a gait [13], although alternative interpretations have been proposed [73].

Coronal plane: In the coronal plane, habitual bipedalism also requires effective balance and dynamic stabilization of the upper body. This is achieved primarily through the hip abductor complex (gluteus medius, gluteus minimus, and tensor fasciae latae), the valgus knee, and the perpendicular orientation of the distal tibial articular surface [12]. Together, these adaptations stabilize the trunk over the supporting limb and reduce lateral “listing” toward the unsupported side [13]. Nevertheless, this stabilization has implications for the IVDs, particularly in the lumbar region. During heel strike, the pelvis tilts approximately 10–15° toward the contralateral unsupported limb, resulting in alternating lateral flexion of the lumbar spine during each gait cycle [74].

Transverse plane: In the transverse plane, habitual bipedal locomotion requires counter-rotation of the upper and lower body during walking and running [12,63]. During each stride, the torso rotates out of phase with the pelvis, such that contralateral upper and lower limbs swing forward simultaneously. This alternating motion helps dampen torsional stresses imposed on IVDs by posterosuperior GRFs generated at heel strike [63]. Because the contacting foot lies lateral to the midsagittal axis, these GRFs tend to twist the pelvis and torso toward the stance limb, subjecting the thoracolumbar spine to alternating torsional loads not experienced by pre-bipedal ancestors. Additional torsional stresses arise from sagittal spinal curvatures, which displace segments from the vertical axis of rotation and may create localized stress concentrations through a cam-like mechanism [75,76].

## 2. Study Objective

The primary objective of this study is to examine key anatomical modifications in human spinal segmental motion that facilitate efficient bipedal locomotion. Specifically, we aim to identify structural differences between humans and non-human primates that reflect functional adaptations to habitual upright posture and load transmission.

## 3. Study Design

This comparative study examines the macro- and microstructural properties of vertebrae and IVDs in humans and apes, focusing on six anatomical components [77]:

The vertebral body (VB),
The intervertebral disc (IVD),
The apophyseal (annular) bony ring (AR),
The annulus fibrosus (AF),
The vertebral endplate (including subchondral bone), and
The vascular structures supplying the IVD.

## 4. Working Hypotheses (H)

### 4.1. Enlargement of the Vertebral Body

H1a.  *The relationship between body mass (kg) and discal surface area in humans will deviate from the scaling pattern observed in non-human primates.*

H1b.  *Vertebral body proportions (length/width/height) will differ significantly between humans and chimpanzees, reflecting substantial enlargement of vertebral body width in humans.*

H1c.  *Vertebral body bone density will be greater in chimpanzees than in humans.*

H1d.  *Cortical shell thickness in humans will be independent of discal surface area and body height (stature).*

H1e.  *Discal surface area in humans will show a strong positive correlation with stature.*

### 4.2. Enlargement of the Intervertebral Disc (IVD)

H2a.  *When standardized to vertebral body height, IVD height will be greater in humans than in chimpanzees.*

H2b.  *In humans, IVD height increases progressively along the cranio-caudal axis, whereas this pattern is not observed in chimpanzees.*

### 4.3. Narrowing of the Apophyseal Ring

H3a.  *Relative to discal surface area, apophyseal (annular) rings will be thinner in humans than in apes.*

### 4.4. Narrow Annulus Fibrosus

H4a.  *Humans will exhibit fewer lamellae in the annulus fibrosus than non-human primates, with the majority of lamellae anchoring to the vertebral endplate rather than to the bony ring, as observed in apes.*

### 4.5. Endplate Thickening and Subchondral Bone Thinning

H5a.  *Humans will exhibit thicker vertebral endplates and thinner subchondral bone than chimpanzees.*

### 4.6. Increased Vascularization at the Vertebral-Endplate Interface

H6a. *Humans will display a greater number of arterial buds at the bone–endplate junction compared with apes.*

## 5. Materials

The samples used to test each working hypothesis are detailed below.

### 5.1. Hypotheses 1b and 3a

To evaluate vertebral body proportions and apophyseal ring dimensions, we examined complete, skeletonized vertebral columns from the Hamann–Todd Osteological Collection (Cleveland Museum of Natural History, Cleveland, Ohio, USA). The sample comprised:

Humans: 240 adult skeletons (120 males and 120 females), ages 20–80 years.

Common chimpanzees (*Pan troglodytes*): 20 adult specimens (9 males and 11 females).

Lowland gorillas (*Gorilla gorilla*): 25 adult specimens (14 males and 11 females).

Additional comparative data were obtained from CT scans of 20 human infants and children aged 2–20 years to assess ontogenetic patterns of discal and vertebral development.

### 5.2. Hypotheses 2a and 2b

The analysis of IVD height and craniocaudal trends was based on a cross-sectional sample of 180 adult humans (males and females) who had undergone clinical CT scanning at Carmel Medical Center, Haifa, Israel. All scans were high-resolution CT images (Brilliance 64 or iCT256, Philips Medical Systems; slice thickness, 0.9–3 mm; 120 kV; 150–570 mA), reviewed using the Extended Brilliance Workspace portal (version 2.36.0.27). This part of the study received ethical approval from the Carmel Medical Center Ethics Committee (CMC-0083-07, approved on 11 November 2008). Comparative CT data for 8 chimpanzees were obtained from the Kyoto University Collection and the Jane Goodall Institute Chimpanzee Eden.

### 5.3. Hypotheses 1a, 1d, and 1e

The evaluation of the relationship between discal surface area, cortical shell thickness, body mass, and stature was based on 210 adult humans (105 males and 105 females) randomly selected from clinical CT records at the Carmel Medical Center (Haifa, Israel). The scanning parameters and analytical procedures were identical to those described above. This component of the study was approved by the Carmel Medical Center Ethics Committee (CMC-0057-11, approved on 21 May 2019).

Comparative vertebral data for non-human primates (n = 38) on vertebral body height and cross-sectional area were obtained from Cotter et al. [25]. Their study examined the eighth thoracic vertebra (T8) from young adult male and female wild-shot gibbons (*Hylobates lar*, n = 10), orangutans (*Pongo pygmaeus* and *Pongo abelii*, n = 8), western lowland gorillas (*Gorilla gorilla*, n = 10), chimpanzees (*Pan troglodytes*, n = 10), and modern humans (*Homo sapiens*, n = 14, 6 male, age 31.3 ± 7.3 years, mean ± SD, range 20–40 years).

### 5.4. Hypotheses 1c, 3b, 4a, 5a, and 6a

Histological analyses of the apophyseal ring, annulus fibrosus, endplate, subchondral bone, and vascular structures were conducted on tissue samples collected from:

- A 48-year-old chimpanzee,
- A 20-year-old orangutan,
- A sub-adult gibbon (exact age unknown), and
- An adult gorilla (age not available)

All apes were from zoological institutions in Israel and Australia and died of natural causes.

## 6. Methods

### 6.1. Hypotheses 1b and 3a

**Figure 1:**
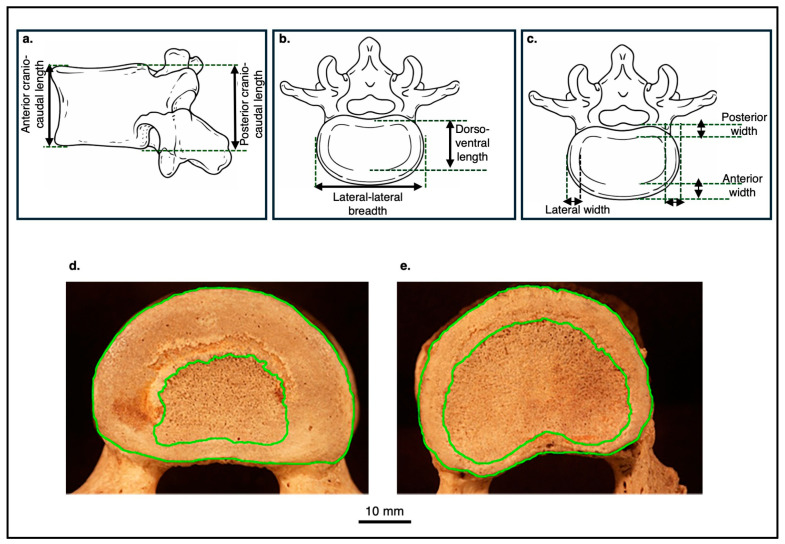
Vertebral body and apophyseal ring measurements: ventral (anterior) and dorsal (posterior) vertebral body craniocaudal length (body height) (**a**); dorsoventral (anteroposterior) vertebral body length and lateral–lateral (right–left) vertebral body breadth (**b**); apophyseal ring ventral, dorsal, and lateral widths (**c**); apophyseal ring area (bordered by green lines) in gorilla (**d**) and humans (**e**).

To evaluate vertebral body size and apophyseal ring dimensions, we examined vertebrae from T4 to L5 in humans and from T4 to L4 in apes. Measurements were taken for both the superior and inferior discal surfaces of each vertebra. The following variables were recorded (Figure 1):

- Vertebral body dimensions: anterior and posterior height (craniocaudal length in apes); discal surface length (anterior–posterior in humans and dorsoventral in apes) and breadth (of both the superior and inferior surfaces; right–left in humans and lateral–lateral in apes).
- Apophyseal ring dimensions: anterior, posterior, and lateral (right and left) ring diameters.

All linear measurements were obtained using a Mitutoyo digital caliper (accuracy ± 0.1 mm).

Each vertebral discal surface was photographed with a digital camera (Minolta Dimage 7, 5.2 megapixels, Minolta Co., Ltd., Osaka, Japan) with an embedded scale. Using ImageJ software v. J1.51, National Institutes of Health (NIH) and the Laboratory for Optical and Computational Instrumentation (LOCI) at the University of Wisconsin-Madison, USA, a boundary (green lines) was traced along the external margin of the discal surface to calculate the total discal surface area. A second boundary was traced along the inner margin of the apophyseal ring (Figure 1). The apophyseal ring area was calculated by subtracting the inner measurement from the total discal surface area [78].

Based on these measurements, the following ratios (indices) were computed:

1. Anterior ring diameter/vertebral body length (anterior–posterior/dorsoventral)
2. Lateral ring diameter/vertebral body breadth (lateral–lateral/right–left)
3. Posterior ring diameter/vertebral body length (anterior–posterior/dorsoventral)
4. Ring area/total discal surface area

Because the vertebral formulae of apes differ from those of humans (apes possess one additional thoracic vertebra and one fewer lumbar vertebra), homologous comparisons were defined as follows: ape T13 ↔ human L1, ape L1 ↔ human L2, and ape L4 ↔ human L5.

### 6.2. Hypotheses 1a, 1d, 1e, 2a, and 2b

Vertebral body height, cortical shell thickness, and IVD height were measured from clinical CT scans of the lumbar region. Height measurements were obtained in the mid-sagittal plane at three locations: anterior, middle, and posterior. Cortical shell thickness was measured on the mid-coronal plane on the left and right vertical walls.

**Figure 2:**
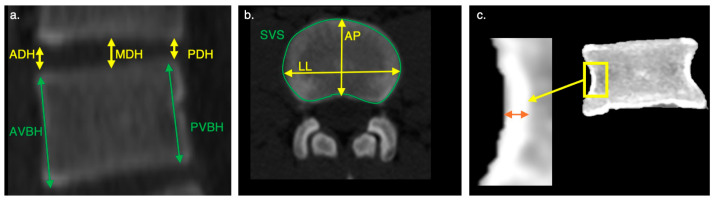
Measurements taken on the vertebrae CT images. ADH = anterior disc height, MDH = middle disc height, PDH = posterior disc height, AVBH = anterior vertebral body height, PVBH = posterior vertebral body height (**a**); AP = anterior–posterior (dorsoventral) discal surface length (body length), LL = lateral–lateral (right–left) discal surface breadth (body breadth), SVS = superior vertebral body surface area (encircled by green line) (**b**); cortical shell thickness (orange arrow), measured on mid-coronal section of the vertebral body at mid-height (yellow box) (**c**).

Intervertebral disc height definitions (Figure 2):

- Anterior disc height (ADH): distance between the anteroinferior and anterosuperior corners of adjacent vertebrae.
- Middle disc height (MDH): distance between the midpoints of the inferior and superior endplates.
- Posterior disc height (PDH): distance between the posteroinferior and posterosuperior corners of adjacent vertebrae.
- The mean disc height was calculated from these three values.

Vertebral body height definitions (Figure 2):

- Anterior (craniocaudal) vertebral body height (AVBH): the distance from the anterosuperior to anteroinferior corner.
- Posterior vertebral body height (PVBH): the distance from the posterosuperior to the posteroinferior corner.
- A mean vertebral body height (MVBH) was calculated from these two values.
- Relative disc height was computed as the ratio between disc height and vertebral body height.

Cortical shell thickness (Figure 2):

- Cortical shell thickness (CST) was measured between the most lateral and the most medial aspect of the vertical wall of the vertebral body, at mid-height, on both the right and left sides.

### 6.3. Hypotheses 1a and 1d

Discal surface areas in humans were measured from CT scans by averaging the superior and inferior discal surface areas for each vertebral body (Figure 2). Comparative data for non-human primates (n = 38) were derived from Cotter et al. [25]. Note that the latter is related to body cross-sectional area, not to discal surface area.

### 6.4. Hypotheses 1c, 3b, 4a, and 5a

For histological analyses, the L3–L4 motion segment was excised from human and ape cadavers. After three weeks of decalcification, the specimens were embedded in paraffin and sectioned sagittally and coronally at 8 μm. Sections were stained with hematoxylin–eosin (H&E) for overall tissue structure and cellular organization, Von Kossa (VK) to identify mineralized areas—or a combination of the two (HE + VK) and Van Gieson (for bone and connective tissues)—and examined under light and polarized light microscopy. These preparations allowed assessment of:

- apophyseal ring width as an attachment area for the AF;
- annulus fibrosus lamellar count, thickness, and organization;
- endplate thickness in the center and periphery;
- subchondral bone structure; and
- arterial buds at the bone–endplate interface.

### 6.5. Reliability of Measurements

To assess intra-observer reliability, three vertebrae from 15 individuals were measured three times by a single investigator (G.D.) under identical conditions, with repeated measurements taken on alternate days. For inter-observer reliability, the same vertebrae were independently measured by two investigators blinded to each other’s results. Intraclass correlation coefficients (ICCs) were computed to evaluate both intra- and inter-observer reliability.

### 6.6. Statistical Analyses

Statistical analyses were conducted using JMP (version 19; SAS Institute Inc., Cary, NC, USA). Descriptive statistics (mean, SD, range) were calculated for all vertebral and IVD variables. Differences in vertebral body parameters and apophyseal ring size among human groups (African American males and females and European American males and females) were evaluated using one-way analysis of variance (ANOVA). Pairwise comparisons were performed using Bonferroni-corrected t-tests. Given the unequal variances between humans and apes (Levene’s test) and substantial differences in sample sizes, group means were compared using Welch’s one-way ANOVA, followed by Games–Howell post hoc tests.

Linear regression analyses were performed to assess (a) whether discal surface and vertebral body volume scale differently with body weight in humans compared with non-human primates and (b) the relationship between discal surface area and vertebral body volume with body dimensions. Height squared and weight raised to the 2/3 power were used to match geometric scaling with discal surface area and ensure linear comparability. All regression analyses were conducted using ordinary least-squares (OLS) estimation. Differences between regression lines were assessed using analysis of covariance (ANCOVA). Multiple regression analysis was performed to identify predictive variables (independent variables: sex, age, body height, body weight, waist circumference, cortical shell thickness) for discal surface area (dependent variable). Because CST measurements derived from CT imaging tend to be larger than those obtained from direct methods (e.g., histology, mCT) [27], we log-transformed the data prior to analysis. Statistical significance was set at *p* < 0.05.

### 6.7. Terminology

In this study, the term “endplate” refers exclusively to the thin hyaline cartilage layer (~1 mm thick) situated between the bony vertebral body and the IVD. This definition excludes the apophyseal (annular) ring from the IVD [79]. In many mammals, the vertebral growth plates resemble mineralized apophyses; the thin hyaline cartilage of the human endplate likely represents the remnant of this ancestral structure.

## 7. Results

### 7.1. Reliability of Measurements

Intra- and inter-observer reliability were high for all variables, with intraclass correlation coefficients (ICCs) exceeding 0.80 in all cases (*p* < 0.001).

### 7.2. Enlargement of the Vertebral Body

H1a.  *Association Between Body Properties (Stature, Weight) and Discal Surface Area and Vertebral Body Volume*.

Data on discal surface area (T7–L5) in humans, chimpanzees, and gorillas appear in Table 1. The human discal surface area is twice that of chimpanzees.

Multiple regression analysis with discal surface area as the dependent variable and sex, age, body weight, body height, waist circumference (at the L3–L4 level), and cortical shell thickness as independent variables yielded a single significant predictor: body height (*p* = 0.044). These results demonstrate that vertebral body enlargement during human evolution was primarily driven by rotational movements, an adaptation consistent with the increased mechanical demands imposed by upright posture and bipedal loading patterns.

**Figure 3:**
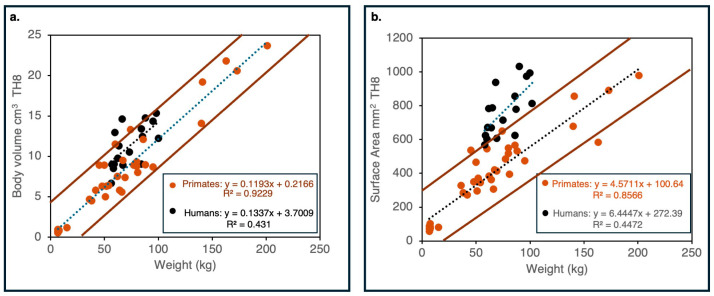
Regression plots of vertebral body volume (**a**) and discal surface area (**b**) of the eighth thoracic vertebra vs. weight in humans (black) and non-human primates (brown). Dotted lines represent the fitted linear regressions for each population (*p* < 0.001). Solid lines indicate the 95% confidence intervals for the estimated regression lines in non-human primates. The human sample (adults only) deviates significantly from the ape’s regression line. Data for non-human primates from Cotter et al. [25]. Note that Cotter and colleagues’ data relate to vertebral body cross-sectional area, not discal surface area, and they calculated body mass from femoral head size, as their study was based on skeletal material.

As shown in Figure 3, the relationship between body mass and discal surface area (mm^2^) in humans differs significantly (*p* < 0.001) from that observed in non-human primates (data for the eighth thoracic vertebra, TH8).

The human discal surface area is substantially larger than expected, given body mass, whereas apes follow a more proportional scaling trend. This results in a significant reduction in the load (weight) borne per unit area of the vertebral surface: on average, humans exhibited 104 g per 1 mm^2^ of discal surface area (SD = 16 g/mm^2^), compared with 140 g per 1 mm^2^ in non-human primates (SD = 44 g/mm^2^). These findings suggest that humans possess disproportionately enlarged discal surfaces relative to body mass, a pattern consistent with the increased mechanical demands associated with upright posture and bipedal locomotion.

H1b.  *Expansion of Vertebral Body Width as the Primary Driver of Human–Ape Differences.*

**Figure 4:**
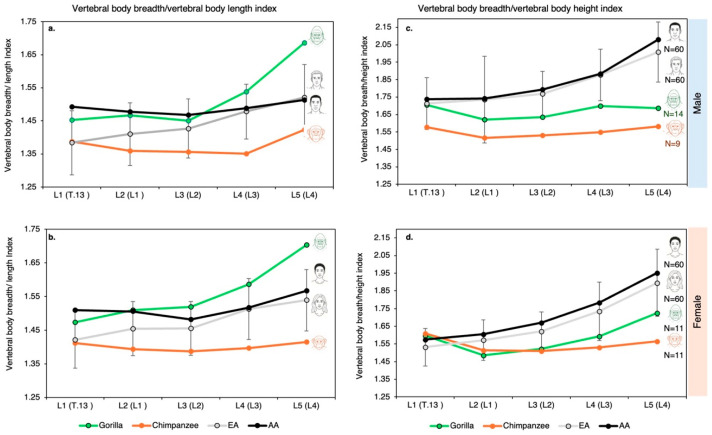
Changes in the ratios of vertebral body breadth to vertebral body length throughout the lumbar spine in African Americans, European Americans, and Gorilla and Chimpanzee males (**a**) and females (**b**) and vertebral body breadth to vertebral body height in African Americans, European Americans, and Gorilla and Chimpanzee males (**c**) and females (**d**). Means are significantly different between all samples (see Supplementary Materials: Welch’s one-way ANOVA). Statistical analysis of individual comparisons (Games–Howell post hoc tests between pairs) appears in the Supplementary Materials Section S1. Data are presented as means, with vertical error bars indicating one standard deviation (±1 SD).

**Figure 5:**
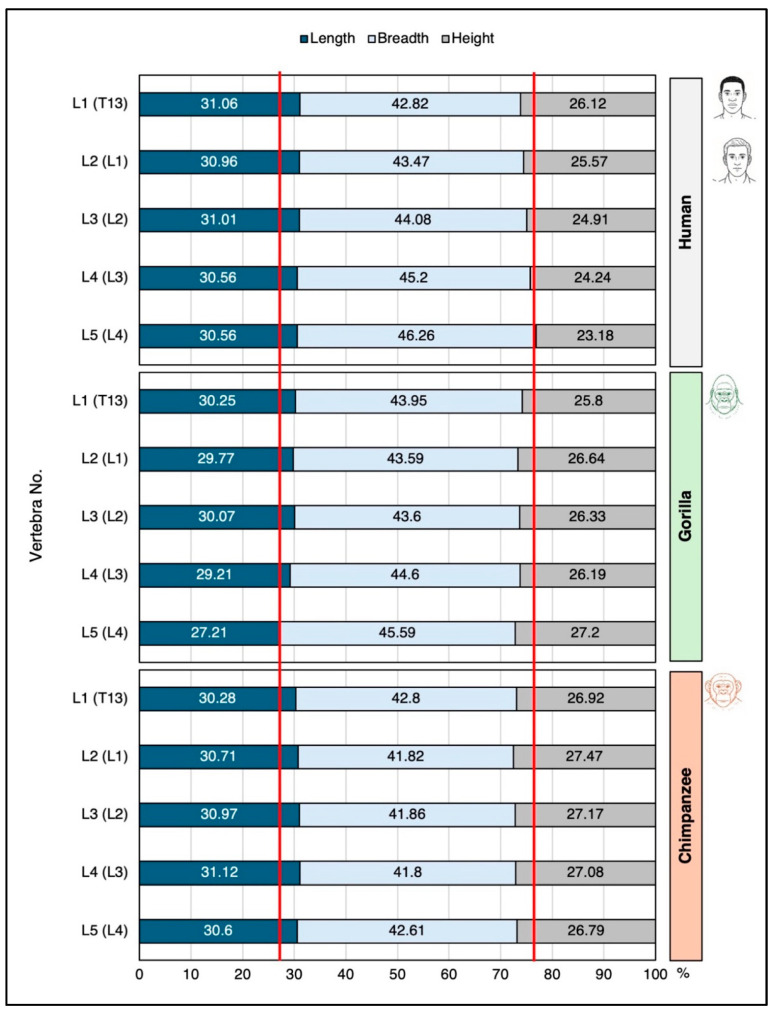
Changes in lumbar vertebral body dimensions in humans, chimpanzees, and gorillas. Numbers (%) reflect the relative contribution of each dimension to the total size of the vertebra (length + breadth + height = 100%). Numbers in parentheses denote the vertebra location in the apes’ spine. The left vertical red line represents the minimal value for the length (AP/DV) dimension and the right line represents the maximal value for the width dimension (LL/RL).

**Figure 6:**
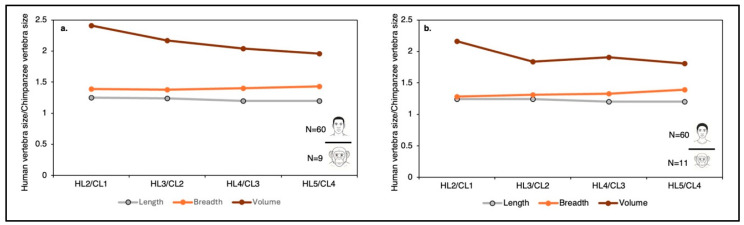
Human-to-chimpanzee ratios of lumbar vertebral body morphology in males (**a**) and females (**b**). Each line represents the ratio between species for a specific dimension: vertebral body length (gray), vertebral body width (orange), and vertebral body volume (red). Values > 1 indicate relatively larger dimensions in humans. H = human, C = chimpanzee, L = vertebra location; numbers indicate vertebra position (e.g., L1 = first lumbar vertebra).

Comparative analysis of lumbar vertebral body dimensions (height, length, and breadth) reveals clear interspecific differences among humans, chimpanzees, and gorillas (Figure 4, Figure 5 and Figure 6). The transition from the chimpanzee condition to the human condition is characterized primarily by a substantial increase in vertebral body breadth.

Vertebral circularity (breadth/length ratio) varies among species (Figure 4 and Supplementary Materials Section S1):

- Gorillas show the largest craniocaudal change in this index, with vertebral bodies becoming progressively more oval in shape along the lumbar spine.
- Chimpanzees exhibit minimal fluctuation across the lumbar region and possess the most circular vertebral bodies.
- Humans fall between the two ape species, although minor population-level differences are observed (e.g., African Americans vs. European Americans).

Breadth-to-height proportions further differentiate humans from African apes (Figure 4 and Supplementary Materials Section S1). In humans, this ratio increases markedly from L1 to L5 (from ~1.7 to ~2.05), whereas in chimpanzees and gorillas, it increases only slightly (from ~1.6 to ~1.7). Thus, human lumbar vertebral bodies are substantially wider relative to height than those of African apes (see Supplementary Materials).

Analysis of the relative contribution of each dimension to overall vertebral body size supports these observations (Figure 5):

- In humans, the anteroposterior (AP) length contribution remains constant throughout the lumbar spine;
- the breadth contribution increases caudally;
- the height contribution decreases.

In contrast:

- Chimpanzees show virtually no change in dimensional contributions from L1 to L5, indicating very limited shape variation.
- Gorillas exhibit slight increases in breadth and height contributions, accompanied by a modest decrease in AP length (Figure 5).

Absolute measurements further highlight these distinctions. Human lumbar vertebral bodies are, on average, ~35% wider and ~25% longer (dorsoventrally) than those of chimpanzees (Figure 6). Overall, the volume of the human lumbar vertebral body is approximately twice that of the chimpanzee (Figure 6).

H1c.  *Vertebral Body Bone Density is Greater in Chimpanzees than in Humans.*

**Figure 7:**
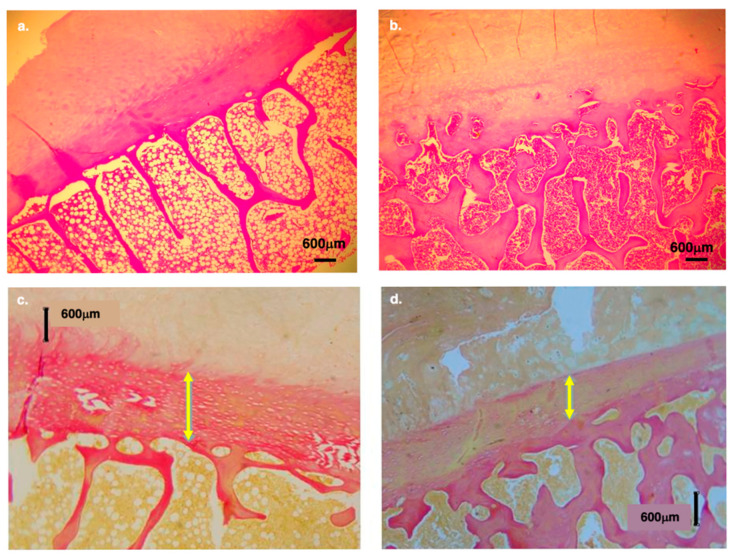
Differences in cancellous (trabecular) bone organization in the vertebral body and endplate thickness of humans and chimpanzees. Human trabeculae are sparse, thin, and generally vertically oriented relative to the discal surface plane (**a**,**c**). The endplate is thick (**c**). Chimpanzee trabeculae are dense, thick, and manifest a maze-like pattern. The end plate is considerably thinner compared to the human condition (**b**,**d**). Vertical arrows mark the boundaries of the end plate in humans (**c**) and chimpanzees (**d**).

Histological examination of lumbar vertebral bodies (Figure 7) reveals pronounced interspecific differences in trabecular architecture. In humans, trabeculae are sparse, thin, and predominantly vertically oriented (relative to the discal surface area), forming column-like structures aligned with the discal loading axis. Horizontal short plates (struts) connect between the longer vertical bony plates (Figure 7a,c). This configuration reflects an adaptation to repetitive axial loading associated with bipedal posture.

In contrast, chimpanzee trabeculae are substantially denser, thicker, and arranged in a complex, labyrinth-like network, with more interconnecting struts and a less purely vertical, more isotropic organization. This highly interconnected trabecular structure is consistent with the elevated mechanical demands imposed by climbing, suspension, and knuckle-walking, where the vertebral column experiences greater multidirectional stresses.

Together, these observations demonstrate that chimpanzees possess markedly higher vertebral body bone density and trabecular robustness than humans, underscoring fundamental mechanical and functional differences in spinal loading regimes across species.

H1d.  *Cortical Shell Thickness is Independent of Discal Surface Area.*

**Figure 8:**
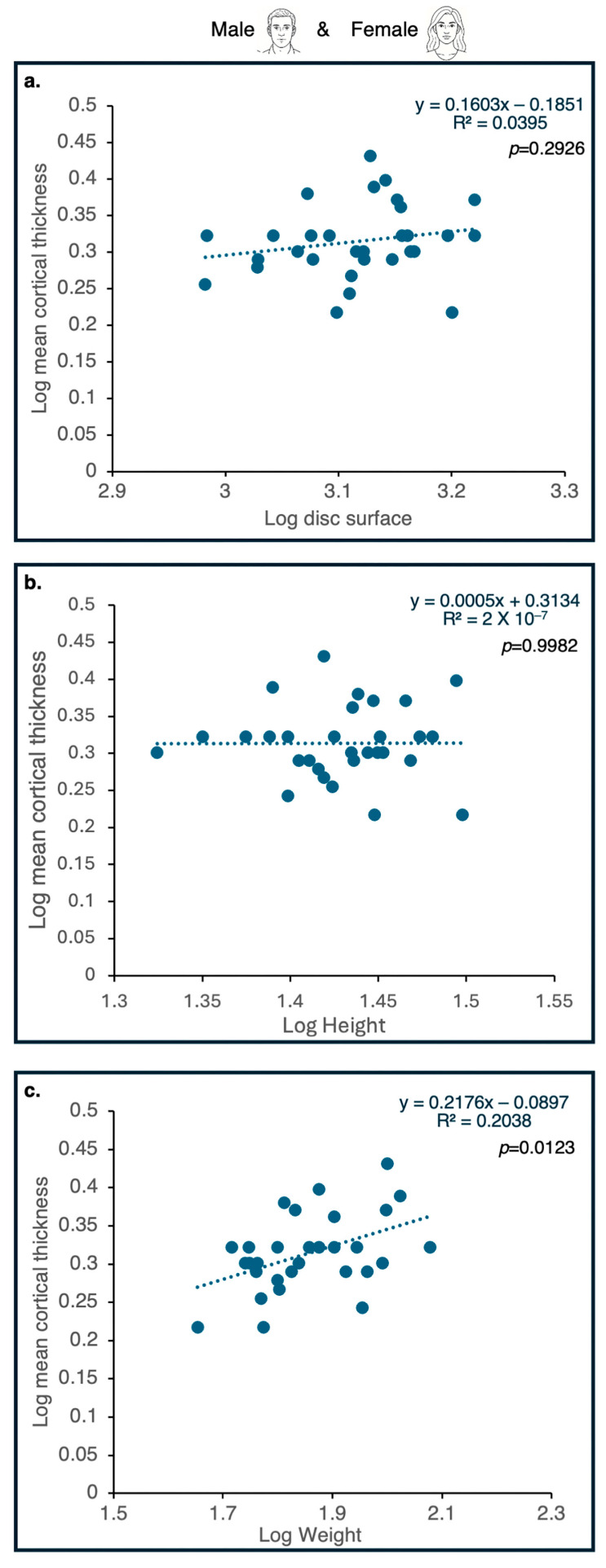
Regression plots of cortical shell thickness of L3 with discal surface area (**a**), body height (**b**), and body weight (**c**). Males and females combined; data were log-transformed. Dotted lines represent the fitted linear regressions.

The results presented in Figure 8 clearly show that, as with the vertebral centrum, the expansion of the human vertebrae was not accompanied by an increase in cortical shell thickness. No significant association was found between this parameter and discal surface area or body height. As in apes, it correlates with body weight.

H1e.  *Discal Surface Area in Humans and Vertebral Body Volume Area are Strongly Correlated with Stature.*

**Figure 9:**
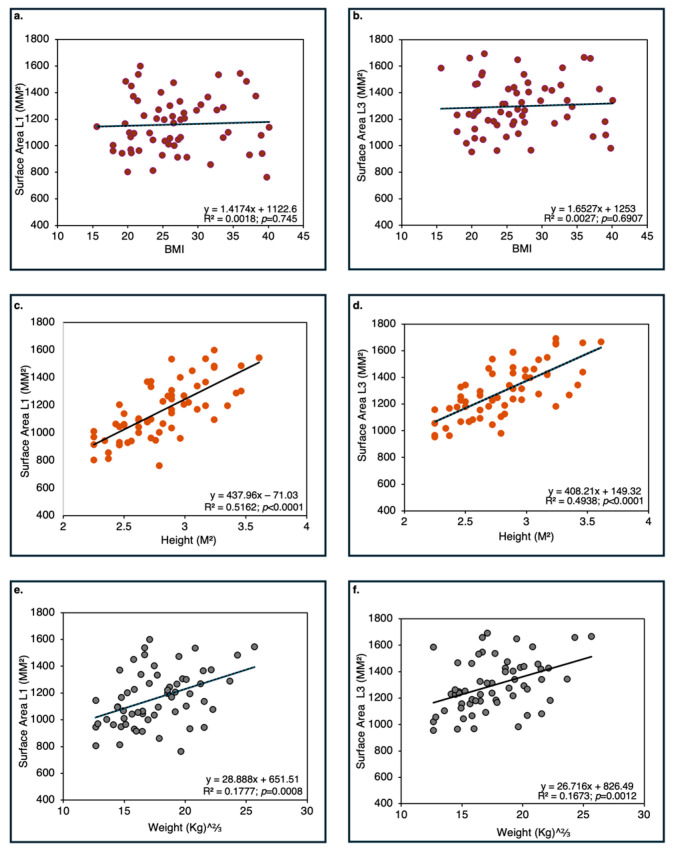
Relationships between discal surface area and body size variables [BMI (dark brown), body height (orange dots), and body weight (gray dots)] in the first (L1: **a**,**c**,**e**) and third (L3: **b**,**d**,**f**) lumbar vertebrae. Body mass is scaled to kg^2/3^ and stature to m^2^. Regression equations and associated significance values are shown. Except for BMI, regressions for body weight and body height are statistically significant, demonstrating robust associations between discal surface area and somatic parameters.

**Figure 10:**
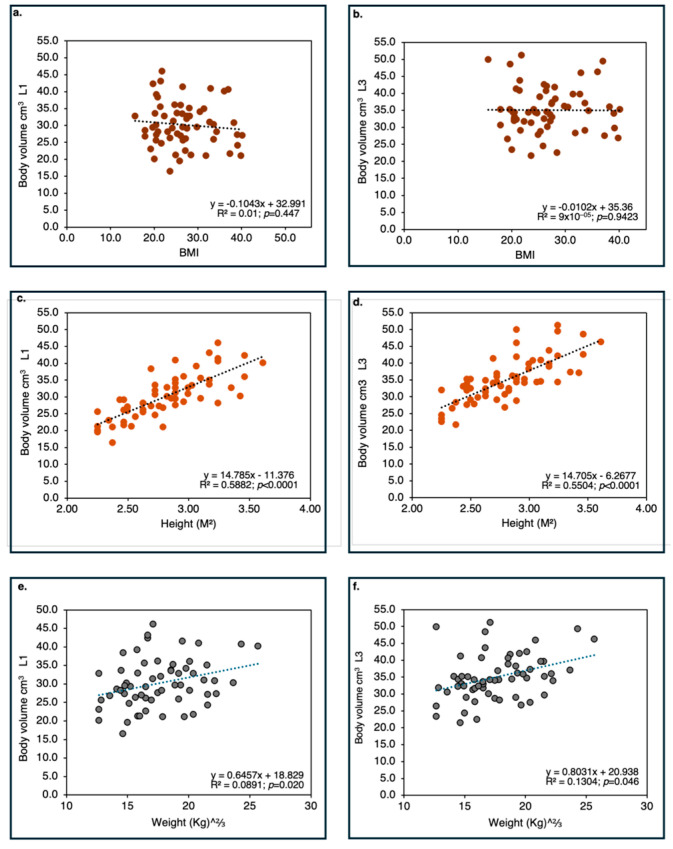
Relationships between vertebral body volume and body size variables {BMI (dark brown), body height (orange dots, and boweight} in the first (L1: **a**,**c**,**e**) and third (L3: **b**,**d**,**f**) lumbar vertebrae. Body mass is scaled to kg^2/3^ and stature to m^2^. Regression equations and associated significance values are shown. Except for BMI, all regressions are statistically significant, demonstrating robust associations between vertebral body volume and somatic parameters.

Across all lumbar levels, discal surface area exhibited significant correlations with stature and body weight, but not with BMI, regardless of sex and vertebra location (Figure 9). Nonetheless, stature (body height) contributed a much larger portion to the explained variance of discal surface area (r^2^ = 0.516) than body weight (r^2^ = 0.177). These results suggest that discal surface enlargement in humans is more closely linked to overall body size, as expressed through linear dimensions, than to body mass alone. When vertebral body volume is considered, stature contributes considerably to the explained variance (55–59%), whereas weight contributes rather little (9–13%) (Figure 10).

### 7.3. Enlargement of the Intervertebral Disc (IVD)

H2a.  *Relative to Vertebral Body Height, IVD Thickness is Greater in Humans than in Chimpanzees.*

**Figure 11:**
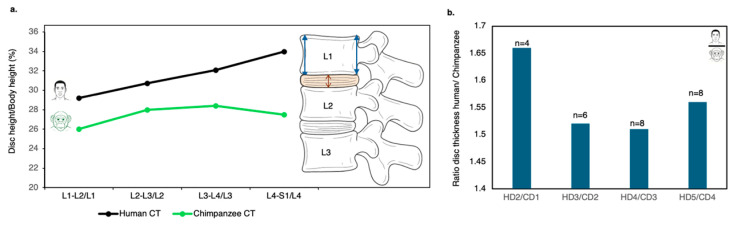
(**a**) Disc thickness (brown arrows) relative to vertebral body height (blue arrows) in humans (black line) and chimpanzees (green line) (a CT-based study). L1–L2 = disc thickness between the first and second lumbar vertebrae. L1 = vertebral body height of the first lumbar vertebra. (**b**) Ratios of disc thickness in humans to disc thickness in chimpanzees. HD2 = human disc height between the second and third lumbar vertebrae; CD1 = chimpanzee disc height between the first and second lumbar vertebrae.

Although the chimpanzee sample was relatively small, the results consistently demonstrated that human IVDs are proportionally thicker than those of chimpanzees across all lumbar motion segments (Figure 11).

On average, IVD height in chimpanzees constitutes approximately one-quarter of vertebral body height (26.1%), whereas in humans, it approaches one-third of vertebral body height (31.9%). This proportional increase highlights a key functional distinction in the human lumbar spine. In absolute terms, disc height is 156% greater in humans than in chimpanzees, an enlargement even more pronounced than the interspecific differences observed in vertebral body width (143%) and vertebral body height (126%) (Figure 11b). Human adult lumbar IVDs are thicker (in the center) than those of chimpanzees, ranging from 6 to 11 mm (mean = 8.5 mm), whereas those of chimpanzees range from 5 to 6.5 mm (mean = 5.8 mm). These findings suggest that IVD expansion represents a significant evolutionary modification of the human spinal motion segment, contributing to increased flexibility and improved load distribution during bipedal locomotion.

H2b.  *Relative to Vertebral Body Height, Disc Thickness Increases Craniocaudally in Humans but not in Chimpanzees.*

The data presented in Figure 11a demonstrate a marked interspecific difference in the craniocaudal trends in IVD height. In chimpanzees, relative disc height remains essentially constant across lumbar levels: 26.0% at L1–L2, 28.0% at L2–L3, 28.4% at L3–L4, and 27.5% at L4–S1. This minimal variation indicates an absence of systematic regional differentiation in disc proportions along the lumbar spine.

In contrast, humans show a progressive increase in relative disc height from cranial to caudal levels: 29.2% at L2–L3, 30.7% at L3–L4, 32.1% at L4–L5, and 34.0% at L5–S1. This pattern reflects a functional gradient along the human lumbar column, consistent with increasing mechanical demands placed on lower lumbar segments during bipedal posture and locomotion.

The data strongly support our hypothesis that the IVD represents a functionally central component of human spinal adaptation. Compared with chimpanzees, human IVDs, particularly in the lumbar region, are thicker, more oval, and show a strong correlation with stature. The vertebral bodies appear to have enlarged in close association with these larger discs. A key outcome of this evolutionary pattern is a reduction in vertebral body bone density in humans compared with chimpanzees (Figure 7), indicating that vertebral enlargement increased disc-supporting capacity rather than mechanical strength. Accordingly, expansion of the vertebral bodies is best interpreted as structurally integrated with changes in IVD size, rather than as a direct adaptation for increased load bearing.

### 7.4. Narrowing of the Apophyseal Bony Ring

H3a.  *Relative to Discal Surface Area, Apophyseal Rings in Humans are Thinner than in African Apes.*

**Figure 12:**
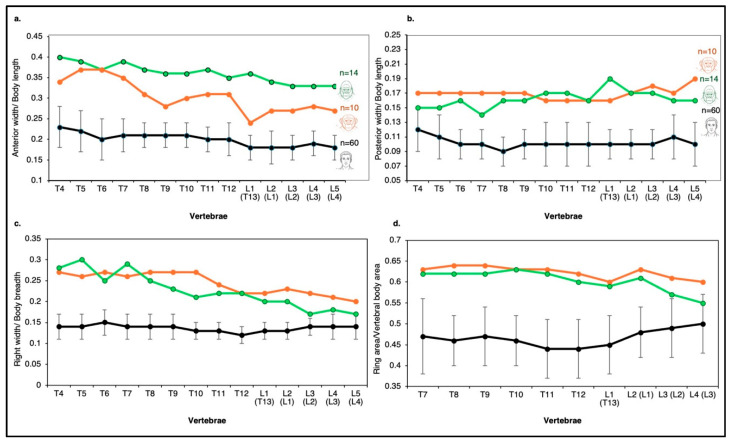
Anterior (**a**) and posterior (**b**) apophyseal ring width relative to vertebral body length (AP/DV), lateral right apophyseal ring width relative to vertebral body breadth (LL/RL) (**c**), and total ring area relative to vertebral body superior discal surface area (**d**) in humans (black), gorillas (green), and chimpanzees (orange). Data for males only. Data are presented as means, with vertical error bars indicating one standard deviation (±1 SD). For detailed statistical analysis, see the Supplementary Materials Section S2. Comparable results were obtained in analyses of the inferior apophyseal rings, confirming that ring narrowing in humans is not restricted to a specific vertebral surface.

Table 2 summarizes apophyseal ring areas across species. Consistently, relative ring widths across all measured vertebrae were narrower in humans than in either African ape (*p* < 0.05; Figure 12a–c, Supplementary Materials Section S2). Figure 12d illustrates the ratio of ring area to superior discal surface area. Welch’s ANOVA revealed significant interspecific differences at all vertebral levels (*p* < 0.01). In every level examined, humans exhibited significantly smaller apophyseal ring areas (relative to vertebral size) than both chimpanzees and gorillas (Figure 12 and associated Supplementary Materials Section S2). In humans, the apophyseal ring occupies, on average, 46.6% of the discal surface, compared with 62.7% in gorillas and 60.3% in chimpanzees. Thus, the apophyseal ring constitutes a substantially smaller proportion of the discal surface in humans than in African apes.

H4a.  *Humans Exhibit Fewer Lamellae in the Annulus Fibrosus.*

**Figure 13:**
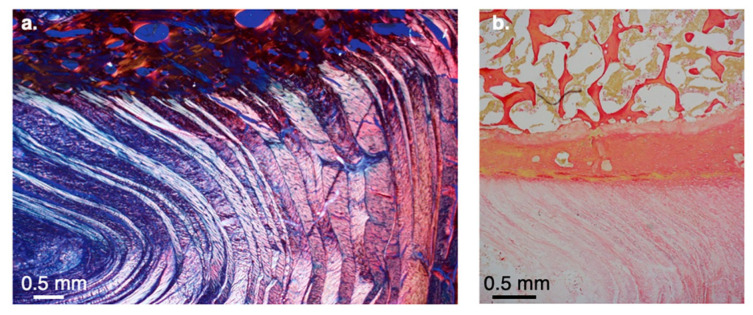
Longitudinal section through the annulus fibrosus of a gibbon (**a**) and a human (**b**) IVD. Notice the complex arrangement and the numerous annuli in gibbons (polarized light). As can be seen, much of the annulus fibrosus in gibbons is inserted directly into bony elements of the discal surface, whereas in humans, most of the annuli are inserted into the hyaline endplate. Note the gradual decrease in thickness of the lamellae from outer to inner in the gibbon.

The number of annular lamellae differs markedly between humans and non-human primates (Figure 13). In humans, the AF typically contains 15–25 lamellae [80], whereas in apes, the small sample studied suggests that the number exceeds 30, indicating a substantially more robust lamellar structure. Histological observations further reveal species-specific differences in lamellar attachment. In gibbons, for example, a large portion of the AF inserts directly into the bony elements of the discal surface (Figure 13). In contrast, in humans, most lamellae insert into the hyaline cartilage endplate, reflecting a different load-transmission configuration and a distinct structural interface between the disc and the vertebral body.

### 7.5. Endplate Thickening and Subchondral Bone Thinning

H5a.  *Humans Possess a Thicker Endplate and Thinner Subchondral Bone and Shell (Lateral Walls) Compared with African Apes.*

**Figure 14:**
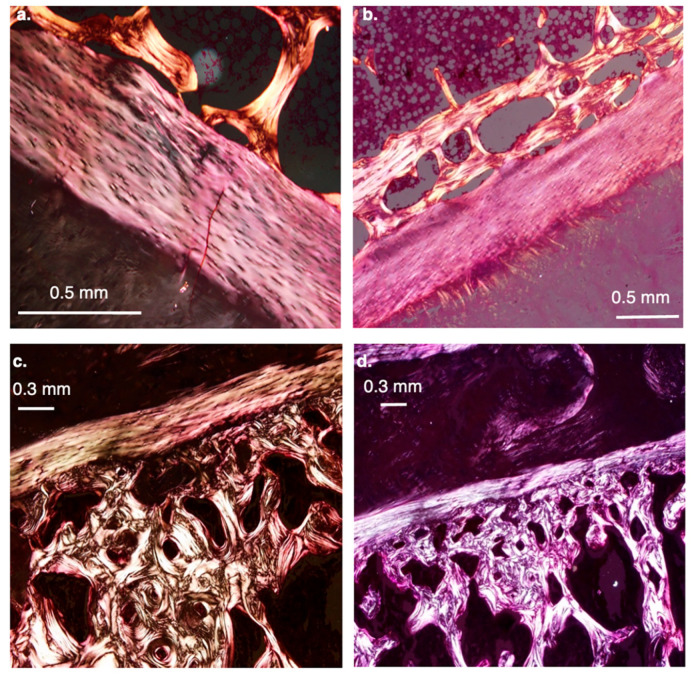
Endplate (hyaline cartilage) and subchondral bone in humans (**a**,**b**) and chimpanzees (**c**,**d**). Note the much thicker endplate and thinner subchondral bone in humans (polarized light microscopy).

Histological analyses reveal that humans have a substantially thicker hyaline cartilage endplate and a thinner, more porous subchondral bone layer than African apes (Figure 14). These differences underscore a fundamental shift in vertebral endplate architecture associated with human spinal loading.

**Figure 15:**
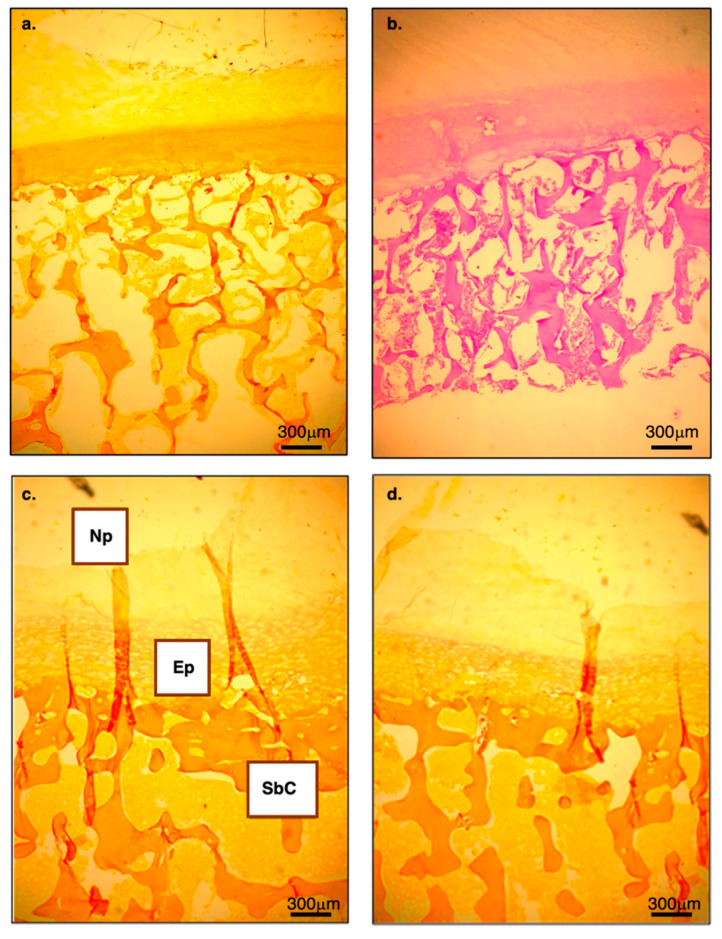
Section through the endplate and subchondral bone. Top: gibbon; endplate is thick relative to subchondral bone. The trabeculae are disorganized, spicule-like, and of various thicknesses (**a**,**b**). Bottom: orangutan; endplate (Ep) is only slightly thicker than the subchondral bone (SbC). Trabeculae are not organized in a recognizable pattern or direction and are thick (**c**,**d**). Np = nucleus pulposus.

In humans, the subchondral region frequently contains double or even triple layers of thin, porous transverse bone (Figure 14b). In contrast, chimpanzees consistently exhibit a single, dense, and solid subchondral plate (Figure 14c,d), indicating a structurally more rigid vertebral interface. These distinctions are also generally observed in Asian apes (e.g., orangutans and gibbons; Figure 15). Based on current data and Cotter et al. [25], shell thickness (mid-height point at the lateral walls of the vertebral body) is, on average, 0.53 mm in humans vs. 0.60 mm in chimpanzees. When scaled by body weight (mass), the difference is even more pronounced (0.0078 mm/1 kg vs. 0.0111 mm/1 kg); that is, the human vertebral bony shell is ~30% thinner than that of chimpanzees.

### 7.6. Increased Vascularization at the Vertebral Endplate Interface

H6a.  *Arterial Buds are Much More Numerous in Humans than in Apes.*

**Figure 16:**
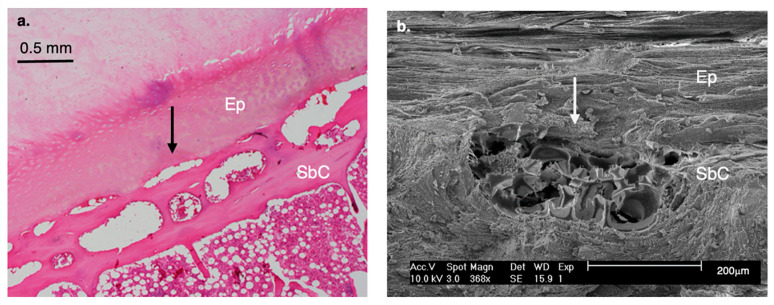
Vascular buds in humans. Left: histological section through the cartilage endplate–bone interface showing numerous blood vessels (marked with black arrow) (**a**). Right: scanning microscopy showing the complex structure of the arterial buds in humans (marked with a white arrow) (**b**). Nutrients diffuse from the vascular buds across the cartilage endplate into the disc itself. Ep = endplate, SbC = subchondral bone. Blood vessels seem to be more abundant in the central region of the disc.

Nutrient delivery to the endplate and IVD (via diffusion) is provided by blood vessels that enter the vertebral body, course through the marrow spaces, and terminate as capillary (arterial) buds (microvascular loops) at the cartilage endplate–bone interface. Histological examination reveals clear interspecific differences in this vascular network (Figure 16).

In humans, arterial buds are numerous, well-developed, and prominently distributed, particularly at the center of the discal surface, as well as in the periphery. This vascular pattern suggests enhanced metabolic support for the disc, consistent with the increased mechanical demands placed on the human spine during habitual bipedalism. In contrast, apes exhibit only sparse arterial buds, reflecting a substantially reduced vascular supply at the vertebral–endplate junction compared with humans. This limited vascularization aligns with their less mobile, mechanically stiffer thoracolumbar spine.

## 8. Discussion

The current discussion is divided into two main sections. In the first section, we discuss the main structural modifications in the human spinal motion segment and their benefits for bipedalism. In the second section, we briefly discuss the implications of these modifications to human health.

### 8.1. Part I: Main Modifications and Their Benefits

#### 8.1.1. Enlargement of the Vertebral Body

Several studies have documented size differences in vertebral bodies between humans and apes, including greater craniocaudal height [26], increased dorsoventral length [19,81], and a progressive craniocaudal increase in mediolateral width [31]. Plomp et al. [21] provide a comprehensive summary table (Table 5 in [21]) of metric traits distinguishing human vertebrae from those of apes. They also discuss the functional implications of several anatomical structures, such as the articular facets, transverse processes, and spinous processes, in relation to the kinematic demands of bipedalism. However, the features examined in that study fall outside the scope of the present analysis.

As shown here, the human lumbar vertebral body volume is more than twice that of the chimpanzee vertebrae. Traditional explanations for the larger vertebrae (relative to body mass) in humans have focused on the greater loading that is presumably associated with orthograde posture. However, this interpretation appears highly unlikely given the reduction in overall bone mass accompanying this expansion, as demonstrated in both the present and previous studies [25]. Indeed, the structural modifications observed in human vertebral bodies (lower bone fraction per volume unit, a thinner cortical shell) suggest that they are less resistant to compressive loads than those of apes [25].

In light of this observation, it appears that the relatively larger sizes of the human vertebral bodies are not due to an increased magnitude of axial loading on the column but rather are a consequence of some other selective factor. As shown in this study, the evolutionary increase in the size of human vertebral elements was likely not driven solely by selection acting directly on vertebral body size but rather reflects strong selective pressures on the spinal motion segment as an integrated functional unit, including the IVD. This conclusion is supported by several lines of evidence. First, the most dramatic interspecific difference between humans and chimpanzees is the thickness of the IVD (156% greater in humans), which substantially exceeds the differences in vertebral body dimensions (143% for width and 126% for height). Second, discal surface area scales strongly with stature (r^2^ = 0.516), suggesting that enlargement of the vertebral body was driven by the need to facilitate extensive rotational movements. Third, human vertebral bodies exhibit reduced bone density, a thin cortical shell, and a lower bone volume fraction relative to body size compared to chimpanzees, as shown in histological analyses, indicating that vertebral enlargement in total discal area did not increase structural strength but rather decreased it. Nonetheless, it reduced the load borne per unit area of the vertebral surface. Fourth, macro and micro structural modifications (e.g., shrinkage of the apophyseal ring, numerous arterial buds, decreased thickness of the subchondral bone) were driven by the necessity to maintain a large and thick IVD. Fifth, ontogenetic analysis of human immature specimens reveals that IVD growth proceeds more rapidly than vertebral body growth during development [82,83,84]. Comparison of juvenile and adult specimens suggests that IVD dimensions increase disproportionately relative to vertebral body size during human development. Sixth, an indirect supporting observation is that the central region of the vertebral body exhibits lower bone mineral density [85] and a lower-quality (more sparse) trabecular architecture [86] compared to the peripheral regions. In this respect, it should be noted that the apparent bone density of lumbar vertebral bodies varies widely (0.05 g/cm^3^ to 0.30 g/cm^3^) between individuals, between levels, and with age [87] and that endplate thickness tends to be thinner in the central region adjacent to the NP [88]. The endplate mean thickness was 0.62 + 0.29 mm [88], yet the IVD increased in thickness from L1–L2 to L5–S1 (~6.5 mm to 9.5 mm), whereas the endplate increased from L1–L2 to L3–L4 (0.50 mm to 0.70 mm) and decreased thereafter (to ~0.6 mm) [88]. According to Bach et al. [89], IVD height increases from L1–L2 (mean = 6.9 mm) to L4–L5 (mean = 9.2 mm) and decreases thereafter at L5–S1 (mean = 8.8 mm). Numbers are smaller for females.

All of the above are consistent with our suggestion that IVD enlargement played a disproportionately important functional role in human spinal evolution and that the larger vertebral body co-evolved as a structural platform capable of supporting a thicker and more mechanically competent IVD.

From a biomechanical perspective, the selective advantage of larger IVDs in bipedal hominins derived from their enhanced ability to: (1) distribute variable, asymmetric loading across a larger endplate surface; (2) absorb energy through greater NP deformation; and (3) resist torsional stresses through increased radius [90,91]. These functional demands are closely associated with the presence of larger vertebral bodies, which merely serve as platforms to support the mechanically enlarged disc.

#### 8.1.2. Enlargement of the IVD

As shown in the current study, the greatest difference in motion segment structure between humans and chimpanzees is the thickness of the disc. The functions of the IVDs are several. By separating the adjoining vertebrae, the IVDs permit motion between the serially arranged bony elements, transforming what would otherwise be a rigid bar-like vertebral column into a flexible series of elements [41,56,92]. They allow the vertebral column to bend and twist [93]. In addition, because the IVDs have a semi-liquid center (the NP) surrounded by a laminated ligamentous ring (the annulus fibrosus), the IVD is able to absorb energy while being compressed through displacement of the nucleus and the bulging of the surrounding ring [94,95]. Importantly, a larger disc is also capable of allowing more angular motion between the adjoining vertebral elements. Larger IVDs also increase energy absorption under compressive loads, and the increase in radius (145% greater in humans compared to chimpanzees) acts to augment the disc’s ability to resist torsional stresses [65]. In addition, the IVDs (mainly NP) have the ability to spread the high compressive forces over a greater area, thus reducing the axial loading per unit [96]. In sum, the IVD functions as a load-bearing fibrocartilaginous joint that redistributes compressive forces, resists shear and torsion, permits controlled motion between vertebrae, and contributes critically to spinal stability during locomotion and posture [41,65,95]. With age, however, the IVD narrows and a significant part of the compressive load is transmitted through the neural arch [97].

#### 8.1.3. Increase in the Transverse Dimension of the Vertebral Body

It is clear that the increase in vertebral body discal surface area was necessary to structurally support a larger disc. However, one question remains: why is the increase in discal surface area more pronounced in the transverse (body breadth) dimension? In other words, why does the shape of the human vertebral body deviate from the chimpanzee pattern?

This probably has to do with balance (equilibrium and stability): while AP balance is achieved by the sinusoidal shape of the vertebral column and its invagination anteriorly (positioning the body’s center of gravity exactly above the geometrical center of the base of support), lateral balance of the upright body, however, is partially achieved by widening the vertebral bodies. This notion is further supported by our findings that discal surface area is highly correlated with stature (51% of the explained variance).

#### 8.1.4. Narrowing of the Annulus Fibrosus

Compared to apes, human annulus fibrosus (AF) is made up of fewer concentric layers (15–25 lamellae) of varied width (0.05–0.5 mm), with increasing thickness from outer to inner [80,98]. The narrowing of the AF observed in humans was probably necessary to tolerate a greater range of torsional movements and to allow the NP to expand, accounting for 40–50% of the volume of the adult disc [99] and 25–50% of the transverse cross-sectional area [45], thereby better absorbing energy and distributing it to surrounding structures. This, however, poses a challenge, as the AF must simultaneously withstand axial loading of the spine (circumferential deformation of the NP under pressure) while enabling large rotational movements between vertebrae [100]. In humans, the AF is structurally optimized to do so through a combination of collagen fiber stretch and interlamellar shearing [98]. This is a unique consequence of its angle-ply organization [101]. In torsion, the collagen fibers in the lamellae oriented in the direction of the twist are stretched, while the fibers in the opposite direction are unloaded [80,102]. In bending, the biaxial fiber layup prevents the disc from buckling on the flexion side while resisting excessive bulging on the extension side. Accompanying overwork and senescence, tears and angular deformities often appear in the apophyseal rings, together with clefting and delamination of the AF. The AF also acts as a meniscus-like pad to transmit compressive forces between adjacent vertebrae. It is not yet clear whether the fact that up to 50% lamellar layers in humans are circumferentially incomplete [98] has to do with the extensive expansion of the human IVD to allow walking and running.

#### 8.1.5. Shrinkage of the Apophyseal Ring

The apophyseal ring in humans’ vertebrae is considerably narrower compared to that in African apes. This unique feature of the vertebra serves two major purposes.

The first is contributing to the development of the sinusoidal spine. Pre-puberty, the osseous rim is separated from the vertebral body by a cartilaginous growth plate. However, at the age of 14 or 15, initial synostosis of the bony annular rim and the vertebral body typically occurs. Complete fusion typically occurs between the ages of 18 and 25 [86,103,104,105,106]. The late fusion of the annular bony ring and the fact that it does not fuse simultaneously up and down the column imply that the final adult morphology is susceptible to “fine-tuning” until complete synostosis. Moreover, depending on their position within the column, the rings begin to fuse at different times along the margins of their respective vertebra. That is, earlier fusion along the lateral sides and posterior margin of the vertebra and somewhat later fusion along the anterior margin (accompanied by continued growth) can result in a posteriorly wedged vertebra. The opposite condition is also true, wherein the posterior margin is delayed, resulting in minor continued growth and an anteriorly wedged vertebra [107].

The second is preserving the vitality of the IVD, the largest avascular structure in the human body [94]. Apart from a sparse blood supply in the outer layers of the annulus, mature discs are almost entirely reliant on diffusion of solutes across the cartilage endplates, mainly in the central region, for nutrition [79,88,108]. Minimizing the area of the discal surface covered by bone enhances diffusion to the IVD. In a previous study of the apophyseal ring [78], we demonstrated that in humans, the absolute width of the apophyseal ring increases in a cephalocaudal direction, paralleling the enlargement of vertebral body size. On average, the ring occupied approximately 47% of the total vertebral body articular (discal surface) area. When normalized to vertebral body surface area, the relative ring area decreased from T4 to T12 and subsequently increased in the lumbar spine (from 48% at L1 to 56% at L5, measured on the inferior surface). We suggested that this pattern reflects increasing mechanical demands on the AF in the lumbar region, including greater compressive loading from upper body weight and increased tensile stresses associated with flexion–extension mobility (the AF in the lower lumbar IVD is thicker, and most laminae are inserted into the bony ring to better stabilize the motion segment).

We further proposed that the greater thickness of the anterior portion of the ring is attributable to higher compressive forces acting on the anterior vertebral body in an erect posture, as the center of gravity lies anterior to the spine. Trabecular architecture indicates that the middle and posterior regions of the vertebral body are better adapted to resist loading than the anterior region [54]; thus, anterior ring widening, corresponding to a thicker AF, may compensate for this relative structural vulnerability. In contrast, the posterior AF experiences reduced tensile stress due to biomechanical and anatomical factors. The axis of sagittal motion lies posterior to the vertebral center and shifts contralaterally during torsion, resulting in greater strain on anterior annular fibers, particularly in the oval-shaped lumbar vertebrae [109]. Additionally, posterior stabilizing structures, including the zygapophysial joints, posterior ligaments, and deep paraspinal muscles, attenuate stress on posterior annular fibers. During flexion, tension in posterior ligaments and facet joint capsules limits motion, whereas during extension, the anterior longitudinal ligament reinforces the highly stressed anterior AF [110]. Although, to a lesser extent, the lateral portions of the epiphyseal ring are also substantially wider than the posterior portion. Because lateral flexion of the spine is resisted primarily by the lateral regions of the IVD, with minimal contribution from the neural arch or surrounding ligaments, a thicker AF is required in these areas. Consequently, the lateral aspects of the AF serve as the principal structures controlling intervertebral motion in the coronal plane. In a previous paper [111], we discussed in detail how the organization and the microstructure of the AF work to retain the hydrostatic pressure of the NP, control the range of motion, and maintain the integrity of the motion segment.

#### 8.1.6. Thickening of the End Plate

Although careful analysis of IVD microstructure could be carried out on a very few non-human primates, the impression is that human endplates are thicker than those in African apes. They are approximately 0.6-mm thick in humans [88,112] and seem to be much thinner in apes. This is not surprising as mechanical loading is a major determinant of endplate thickness [113]. Additionally, it gives more flexibility to the human IVD and serves as an anchorage site to the inner lamellae of the AF [114]. Also, the endplate is thinner at its center, likely to facilitate nutrient diffusion to the NP [88]. It increases in size in the transverse plane, following the enlargement of the vertebral bodies [115]. The endplate is bonded weakly to the underlying bony layer.

#### 8.1.7. Double-Layer Subchondral Bone and Organization of the Trabecular Bone

Although thinner and more porous than that of African apes, human subchondral bone (sometimes called bony endplate) often exhibits double or triple-spaced horizontal bony layers. Studies have shown that it is stiffest and strongest around the periphery of the VB [116]. The general reduction in bone mass per volume unit considerably weakens the human vertebral bodies, exposing them to mechanical stresses (vertebral body collapse) and pathological conditions (invagination of the NP into the vertebral body, i.e., Schmorl’s nodes).

The double layer of bony plate beneath the endplate, commonly observed in human vertebrae, appears to provide additional support to the IVD by enhancing its ability to withstand loads applied to the vertebral body surface. This structural feature may also contribute to greater resistance to strain-induced deformation (due to its greater flexibility).

The vertebral body is composed primarily of cancellous (trabecular) bone organized into a highly ordered, load-adapted lattice. As shown in this study, the trabeculae form intersecting vertical and horizontal struts: thick, plate-like vertical trabeculae align with the principal compressive forces transmitted along the spinal axis, while thinner transverse trabeculae interconnect them, providing structural stability and resistance to shear. This anisotropic architecture efficiently distributes mechanical loads from the IVDs to the cortical shell, maximizing strength while minimizing mass. The organization of cancellous bone within the vertebral body undergoes continuous remodeling in response to habitual loading, making its pattern a sensitive indicator of the biomechanical environment, age, and pathology.

#### 8.1.8. Multiple Arterial Buds

Humans exhibit an increasing number of blood vessels/arterial buds (which are more densely located at the center of the discal surface) at the bony-endplate interface compared to African apes. This modification was necessary to ensure adequate nutrition of the IVD, the largest avascular structure in the human body.

#### 8.1.9. Thin Cortical Shell

Lumbar vertebral cortical shell thickness ranges, based on direct measurement, between ~0.25 and 0.45 mm [27]. Because clinical CT overestimates shell thickness (due to segmentation and resolution effects) [27], we had to log-transform our data and rely on the literature for its actual thickness. Nonetheless, the literature generally agrees that the shell is thin (rarely exceeding 0.5 mm) and porous [27,117,118]. Why did the shell remain thin? Was this not associated with discal surface area and body height, as shown in the current study? We think this thin cortical shell architecture likely represents an adaptive trade-off: despite its thinness, the shell plays a disproportionate mechanical role by enclosing the trabecular centrum and directly transmitting loads from the IVDs. Finite-element and experimental studies [117] show that this shell contributes substantially to vertebral stiffness and strength, particularly under non-axial loading. In habitual bipeds, lumbar vertebrae experience cyclic compression, torsion, and lateral bending, placing alternating stresses on the cortical shell that were minimal in pronograde ancestors. This thin-shell architecture, allowing elastic deformation during repetitive loading, likely represents an adaptive compromise, permitting flexibility and mobility while increasing sensitivity to fatigue and stress concentration at curvature apices and transitional zones. It therefore seems evident that the human lumbar spine achieves bipedal flexibility not by reinforcing the vertebrae with thick cortex but by relying on an exceptionally thin cortical shell that bears complex, repetitive loads from the IVDs.

In sum, while the IVD macro- and microstructure of non-human primates is highly comparable to that of humans in terms of collagen organization, proteoglycan distribution, and basic histological features [119,120,121], our results suggest that bipedal loading has driven detectable modifications at the two levels to ensure a vivid, mechanically functioning IVD. As peak vertical GRFs at heel strike are approximately twice as high during running as during walking and may approach 3–4 times at higher endurance running speeds [122], lateral and vertical expansion of the IVD (disproportionally to body weight) was instrumental (to reduce stresses) in enabling sustained long-distance running, a derived capability of the genus Homo [12]. The expanded human IVDs also provide greater rotational movement within segmental motions and increased trunk stabilization, both of which are necessary for walking and running.

### 8.2. Part II: Main Modifications: Implications for Human Health

Most modifications in the spinal motion segments reported in this study (e.g., larger vertebral bodies, thinner apophyseal ring) are associated with thick IVDs. The structure and size of the human IVD suggest that the spinal column enhances balance and flexibility at the expense of energy absorption capabilities. Indeed, torsional forces are significantly greater in human spines than in those of apes [8,13,40,123,124], which probably explains why radial tears are common in humans and almost absent in non-human primates [125]. The increased spinal mobility in humans is achieved through fewer, but longer, annular fibers, as well as a larger NP. Furthermore, the upright bipedal posture subjects the vertebral column to highly repetitive, diverse types of off-axis, unpredictable loading, much of which is due to the single-limb support phase. To better direct and control these potentially damaging forces, humans invaginated the spine into the thorax and increased vertebral body size to support the large IVD. This, in turn, led to a relative thinning of the apophyseal ring, the loss of cortical (thin-shell) and more spaced cancellous bone, and a thicker endplate (a remnant of the original growth plate). Because the ability to directionally constrain and absorb intervertebral forces depends on the muscular envelope surrounding the thoracolumbar spine, any perturbations in this system (due to tissue fatigue and age-related damage or novel loading scenarios such as carrying objects) can result in damaging levels of stress. Unfortunately, in the face of insufficient muscular strength and muscle fatigue and/or novel motions that do not permit normal eccentric dissipation of loading, the thinner AF, expanded nucleus pulposus, thinner endplate in the central region, and reduced bone density can result in disc herniations and/or vertebral fractures. Age-related degenerative changes in IVD components [126], such as delamination of the annular ring and increased stiffness of the NP, also contribute to this scenario.

Compressive overload primarily affects the subchondral bone, and vertical extrusion of the NP may result in the formation of Schmorl’s nodes [127]. Given the high prevalence of disc herniation in humans [128,129,130], it is reasonable to ask why this condition is so common in our species. The explanation lies in both our evolutionary history and our modern cultural environment. From a biological perspective, the vertebral column was not engineered de novo for bipedalism. Rather, it represents a structure incrementally modified from a quadrupedal ancestor under multiple functional constraints. As such, the human spine is best understood as a historically constrained compromise, one that permits efficient upright locomotion while simultaneously increasing susceptibility to mechanical stress, degeneration, and structural failure. From a cultural perspective, modern lifestyle factors impose mechanical demands that differ substantially from those experienced by our ancestors. Increased longevity, prolonged sitting, and reduced physical activity expose the spine to loading regimes for which it was not evolutionarily optimized. These factors contribute to the high frequency of IVD failure [131,132] and the widespread occurrence of low back pain [133]. The musculoskeletal system that once protected the spine under ancestral conditions is now challenged by patterns of use and durations of life span for which it was never selected.

It should therefore be emphasized that the costs associated with the human spine do not reflect poor design. Rather, they represent the inevitable outcome of evolutionary trade-offs interacting with novel environmental pressures. In this sense, spinal adaptation to habitual bipedality is not optimal but instead reflects an evolutionarily “good enough” solution.

### 8.3. Alternative Hypotheses

It is plausible that not all observed micro- and macrostructural differences between humans and non-human primates reported here can be unambiguously interpreted as direct adaptations to bipedalism. Alternative evolutionary mechanisms, including genetic drift, developmental constraints, and allometric scaling relationships, may have contributed to some aspects of the observed variation. We therefore acknowledge that not every difference in motion segment structure necessarily reflects direct functional optimization, and that some features may represent byproducts of phylogenetic history, scaling effects, or structural integration [134,135].

That said, the transition to obligatory bipedalism represents one of the most profound biomechanical and postural shifts in human evolution. This shift entailed well-documented system-wide modifications of the axial skeleton. The spine, particularly its load-bearing and motion-segment components, would have been subject to sustained, directionally selective pressures associated with upright posture, habitual lordosis, and altered patterns of axial loading. Within this biomechanical context, it is reasonable to interpret consistent and functionally coherent differences in segmental motion and micro-structural organization as being at least partly shaped by selection related to bipedal locomotion, even if additional mechanisms contributed to their emergence.

We fully acknowledge that the hypothesis that selection acted primarily on the IVD cannot be conclusively tested using extant taxa alone, given the absence of fossil evidence for soft tissues. Accordingly, we frame this hypothesis cautiously, presenting it as a plausible yet currently unresolvable interpretation that is consistent with established biomechanical demands rather than as a definitive evolutionary pathway. Our intention was not to exclude non-adaptive explanations but to propose a functional framework that integrates comparative data from living taxa with established principles of spinal biomechanics, while recognizing the inherent limitations of the available evidence.

### 8.4. Study Limitations

The sample size of non-human primates for some observations is very small (mainly those conducted on CT scans, such as IVD size). The microstructural characteristics of the disc in apes (histological analysis) were determined based on four apes of different species. We, therefore, could only look for qualitative differences between humans and non-human primates. Future studies on a larger controlled (for sex and age) sample are needed to validate our initial observation and gain a deeper understanding of the variability in non-human primates regarding the size and special organization of structures composing the IVD (e.g., endplate thickness). Because our analysis is cross-sectional and comparative, it cannot resolve the temporal sequence of vertebral and disc evolution; however, the strong functional signal observed in IVD morphology highlights its likely importance in human spinal adaptation.

Most of the traits examined in this study have been interpreted as potential adaptations to bipedalism. In the relevant sections, this interpretation is discussed, and the possible functional association with bipedal posture and locomotion is evaluated. However, this discussion should not be understood as an unqualified endorsement of an adaptationist framework. Not all proposed hypotheses have been tested through rigorous comparative analyses that account for phylogenetic relatedness, and none have been examined experimentally. Accordingly, these interpretations should be regarded as provisional. The mere presence of distinctive vertebral traits in humans does not, by itself, establish that they evolved as direct adaptations to bipedalism; alternative explanations, including phylogenetic inheritance, developmental constraints, and non-adaptive by-products, also warrant careful consideration.

### 8.5. Summary

The principal macro- and microarchitectural differences in the components of the spinal motion segments in humans and apes are listed in Table 3. In humans, relatively large intervertebral discs (IVDs) combined with a comparatively thin annulus fibrosus (AF) confer important mechanical advantages, primarily by enhancing the capacity to absorb energy through controlled deformation [11]. Increased disc diameter and flexibility improve resistance to torsional stresses and facilitate the redistribution and redirection of highly variable, asymmetrical loads across the vertebral endplate. The enlargement of both the IVDs and the vertebral bodies likely co-occurred with the partial invagination of the human vertebral column into the thorax, a structural configuration that may further reduce mechanical stress on individual vertebral elements.

Because the IVD is largely avascular and lacks a direct blood supply, its metabolic demands depend on diffusion of nutrients through the adjacent vertebral endplate. Consequently, an increase in disc size necessitates a proportionally expanded bone–endplate interface. This appears to be facilitated by narrowing of the apophyseal ring and the presence of numerous arterial buds, which enhance nutrient diffusion from the vertebral body into the disc, particularly toward the nucleus pulposus.

## 9. Conclusions

The present study offers a novel perspective on the determinants of human spinal architecture. While axial loading is traditionally viewed as the principal force shaping the spine, our findings suggest that the alternating and cyclical forces generated during bipedal walking and running play a dominant role. In humans, the upper limb no longer contributes to the dissipation of locomotor energy, and ground-reaction forces are transmitted through a single supporting limb at any given moment. This biomechanical regime necessitated the evolution of a thick, expansive IVD and a spine composed of alternating curvatures, which together function as an integrated and highly efficient shock-absorbing system.

## Figures and Tables

**Table 1:** Superior vertebral discal surface area (cm^2^) in humans, chimpanzees, and gorillas (males only).

Vertebra	Discal Surface Area (cm^2^) (Mean ± SD)		
Number	Human	Gorilla	Chimpanzee
T7	7.02 ± 0.85	7.34 ± 1.3	3.47 ± 0.42
T8	8.14 ± 0.90	7.82 ± 1.43	3.67 ± 0.39
T9	8.77 ± 1.09	8.83 ± 1.33	4.08 ± 0.26
T10	9.44 ± 1.23	9.06 ± 1.22	4.4 ± 0.35
T11	10.89 ± 1.67	10.16 ± 1.50	4.94 ± 0.36
T12	12.19 ± 1.63	11.62 ± 1.82	5.71 ± 0.62
L1 (T13)	13.03 ± 1.72	13.01 ± 1.97	6.4 ± 0.67
L2 (L1)	13.79 ± 1.60	15.21 ± 2.14	7.21 ± 0.74
L3 (L2)	14.59 ± 1.77	15.88 ± 2.27	7.85 ± 0.81
L4 (L3)	15.29 ± 1.83	16.65 ± 1.91	8.65 ± 1.14

**Table 2:** Superior apophyseal ring areas (cm^2^) in humans, chimpanzees, and gorillas (males only).

Vertebra Number	Apophyseal Ring Area (cm^2^) (Mean ± SD)		
	Human	Gorilla	Chimpanzee
T7	3.34 ± 0.68	4.71 ± 0.60	2.32 ± 0.53
T8	3.79 ± 0.61	5.08 ± 0.73	2.51 ± 0.65
T9	4.14 ± 0.66	5.93 ± 0.91	2.67 ± 0.63
T10	4.4 ± 0.72	5.93 ± 0.91	2.75 ± 0.66
T11	4.87 ± 1.06	6.78 ± 1.06	2.86 ± 0.75
T12	5.42 ± 1.25	7.36 ± 1.30	3.41 ± 0.96
L1 (T13)	5.96 ± 1.10	8.08 ± 1.72	3.69 ± 0.81
L2 (L1)	6.67 ± 1.18	9.83 ± 1.20	4.22 ± 0.98
L3 (L2)	7.3 ± 1.51	10.02 ± 1.44	4.19 ± 0.74
L4 (L3)	7.67 ± 1.57	10.24 ± 1.26	4.46 ± 1.01

**Table 3:** Major macro- and microstructural differences between the human and ape vertebral body, intervertebral disc, and endplate.

Spine Element	Characteristics	Humans	Apes
Vertebral bodies	Bone volume fraction	Low	High
	Size	Large broad bodies	Smaller, more rounded vertebral bodies
	Shape	Trapezoidal (width of superior discal surface < width of inferior discal surface), wedged (anteroposterior) lumbar bodies, presence of pronounced, narrow waist (mid area narrower than superior and inferior areas)	Cylinder-like, no or slight wedging, mid-waist less marked
	Discal surface shape	Oval, heart-shaped	Round/oval
	Shell	Thin	Thick
	Subchondral bone	Thin	Thick
	Trabeculae	Thin	Thick
		Vertically oriented with interconnecting struts	Mesh-like
		The central region of the vertebral body exhibits lower density and a sparser microstructure compared to the peripheral regions, with the highest density posteriorly	Density is similar in all regions of the body
Intervertebral disc	Size	Large relative to body weight Correlate with stature	Correlate with body weight
	Thickness	Thicker lumbar discs	Thinner lumbar discs
	Shape	Wedge-shaped in the lumbar region	More uniform along the lumbar spine
	Apophyseal bony ring	Narrow	Wide
		The anterior part is wider than the lateral and posterior parts, the posterior being the narrowest Increase in width from vertebra L1 to L4	Similar pattern, less marked differences between parts Decrease in width from vertebra L1 to L3
	Annulus fibrosus	Thin (fewer yet longer lamellae)	Thick
		Inserted into the bony (apophyseal) ring and mostly into the cartilage endplate	Most are inserted into the bony ring
Endplate (Hyaline cartilage only)	Size	Thick	Thin
	Area	Covers ~62% of discal surface area	Covers ~47% of discal surface area
	Thickness	Thin in the middle, thick in the periphery	More uniform in thickness throughout
	Chondrocytes	Flat	Rounded
	Blood supply	Numerous arterial buds	Fewer arterial buds

## Data Availability

Detailed statistical analysis supporting the data in the manuscript is reported in the Supplementary Materials.

## Supplementary Materials

The following supporting information can be downloaded at https://www.mdpi.com/article/10.3390/life16030466/s1, Figure S1: Measurements and indices analyzed include vertebral body breadth (LL = lateral-lateral breadth, similar to RL = right-left breadth; Figure 1b) relative to vertebral body length [dorso-ventral (DV) length in apes and anterior-posterior (AP) length in humans; Figure 1b), and vertebral body breadth (LL/RL; Figure 1b) relative to average vertebral body height (AVBH = [Anterior (cranio-caudal length = CC) height + posterior (CC length) height]/2; Figure 1a), in African American (AA) and European American humans (EA), gorillas, and chimpanzees, analyzed separately by sex; Supplemantary Materials Section S1 Statistics for Figure 4. The four graphs show the significance of changes in the ratio of vertebral body breadth to vertebral body length along the lumbar spine in African American, European American, gorilla, and chimpanzee males (a) and females (b), and in the ratio of vertebral body breadth to average vertebral body height in African American, European American, gorilla, and chimpanzee males (c) and females (d). Mean values differ significantly (*) among all groups (Welch’s one-way ANOVA). Pairwise comparisons by vertebra and species are presented below the one-way ANOVA results, using Games-Howell post hoc tests (Welch’s test; JMP v.19); Supplemantary Materials Section S2 Statistical analyses for Figure 12: Anterior (a) and posterior (b) apophyseal ring width relative to vertebral body length (AP/DV), lateral right apophyseal ring width relative to vertebral body breadth (LL/RL), (c), and total ring area relative to vertebral body superior discal surface area (d) in humans, gorillas, and chimpanzees. Data for males only (ordered difference report and Welch’s Test).
