# Supplementary material for: Evolutionary Specializations of the Human Vertebral Body and Intervertebral Disc in Relation to Bipedalism
Source: Life (Basel). 2026 Mar 12;16(3):466. doi: 10.3390/life16030466 (PMC13028001; doi:10.3390/life16030466)
Supplement: Supplementary file 1 [file life-16-00466-s001.zip › life-4097779-supplementary.pdf]

life

Supplementary Materials

Evolutionary Specializations of the Human Vertebral Body and Intervertebral Disc in Relation to Bipedalism

Israel Hershkovitz [1,2,*], Bruce Latimer [3,†], Janan Abbas [4], Mila Hejja [1], Bahaa Medlej [1,5], Hanan Rapoport [1,2], Einat Kedar [1], David Ezra [6], Ian Rybak [1,2], Tatiana Sella Tunis [7], Irit Zohar [8,9], and Gali Dar [10]

1 Department of Anatomy and Anthropology, Gray Faculty of Medical and Health Sciences, Tel Aviv University, Tel Aviv 6997801, Israel
2 The Shmunis Family Anthropology Institute, Dan David Center for Human Evolution and Biohistory Research, Tel Aviv University, Tel Aviv 6997801, Israel
3 Department of Physical Anthropology and Orthopedic Surgery, School of Medicine, Case Western Reserve University, Cleveland, OH 44106, USA
4 Department of Physical Therapy, Zefat Academic College, Zefat 1320611, Israel; janan1705@gmail.com
5 Department of Anatomy, Faculty of Health and Medical Sciences, Arab American University of Palestine, Ramalla 00970, Palestine
6 School of Nursing Sciences, Academic College of Tel Aviv-Jaffo, Jaffo 6818211, Israel
7 Department of Orthodontics, The Maurice and Gabriela Goldschleger School of Dental Medicine, Gray Faculty of Medical and Health Sciences, Tel Aviv University, Tel Aviv 6997801, Israel; tanya.tuniss@gmail.com
8 Department of Biology and Environment, University of Haifa, Oranim 3604301, Israel; zoharir@tauex.tau.ac.il
9 Marine Biodiversity Center, The Steinhardt Museum of Natural History, Tel Aviv University, Tel Aviv 6997801, Israel
10 Department of Physical Therapy, Faculty of Social Welfare & Health Sciences, University of Haifa, Mount Carmel, Haifa 3103301, Israel; gdar@univ.haifa.ac.il

* Correspondence: anatom2@tauex.tau.ac.il; Tel.: +972-36409495
† Deceased prior to manuscript submission.

Supplemantary Materials Section S1

Statistics for Figure 4. The four graphs show the significance of changes in the ratio of vertebral body breadth to vertebral body length along the lumbar spine in African American, European American, gorilla, and chimpanzee males (a) and females (b), and in the ratio of vertebral body breadth to average vertebral body height in African American, European American, gorilla, and chimpanzee males (c) and females (d). Mean values differ significantly (*) among all groups (Welch's one-way ANOVA). Pairwise comparisons by vertebra and species are presented below the one-way ANOVA results, using Games-Howell post hoc tests (Welch's test; JMP v.19).

Figure S1. Measurements and indices analyzed include vertebral body breadth (LL= lateral-lateral breadth, similar to RL=right-left breadth; Fig. 1b) relative to vertebral body length [dorso-ventral (DV) length in apes and anterior-posterior (AP) length in humans; Fig. 1b), and vertebral body breadth (LL/RL; Fig. 1b) relative to average vertebral body height (AVBH=[Anterior (cranio-caudal length=CC) height + posterior (CC length) height]/2; Fig. 1a), in African American (AA) and European American humans (EA), gorillas, and chimpanzees, analyzed separately by sex.

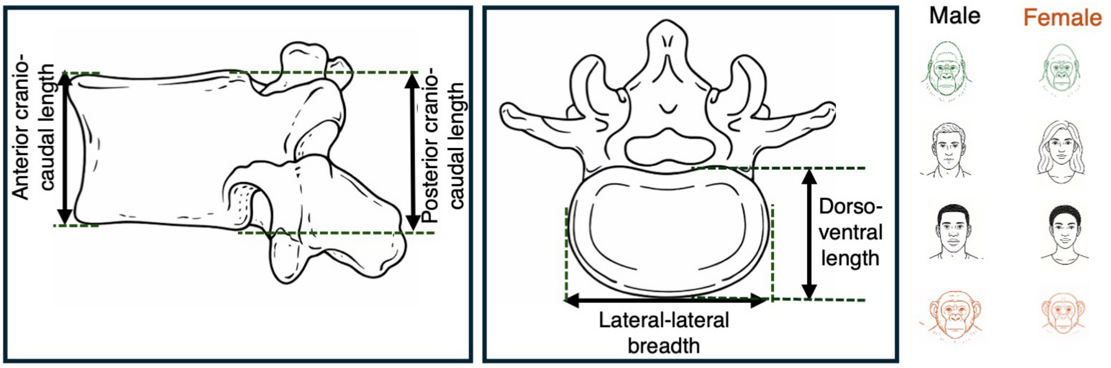

<u>**Abbreviations**</u>

AA – African-American

EA – European-American

M – males

F – females

Chimp – chimpanzee

Gorilla – gorilla

A – anterior

P - posterior

AP – anterior-posterior

DV – dorso-ventral

CC – cranio-caudal

LL – lateral-lateral

RL – right-left

AVBH – average vertebral body height

Vertebra L1 (T13) Male (Figure 4a): Vertebra body breadth (LL/RL)/ vertebral body length (AP/DV)

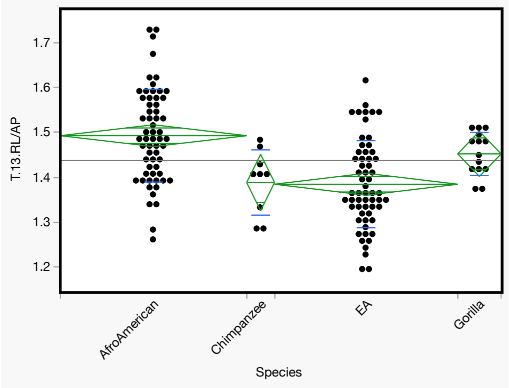

Analysis of Variance:

Source	DF	Sum of Squares	Mean Square	F Ratio	Prob > F
Species	3	0.3759704	0.125323	13.7470	<.0001
Error	138	1.2580623	0.009116		
C. Total	141	1.6340327			

Ordered Differences Report

Species-Male		Differ-ence	Std Err Dif	DF	q*	Lower 95%	Upper 95%	p-Value
AA	EA	0.1086	0.0130	116.7544	2.6064	0.0605	0.1567	<.0001*
AA	Chimp	0.1047	0.0196	13.4253	2.9224	0.0236	0.1858	0.0104*
Gorilla	EA	0.0679	0.0128	41.7332	2.6757	0.0196	0.1162	0.0028*
Gorilla	Chimp	0.0640	0.0194	12.5020	2.9512	-0.0171	0.1452	0.1438
AA	Gorilla	0.0407	0.0131	44.8591	2.6680	-0.0089	0.0902	0.1414
Chimp	EA	0.0039	0.0194	12.8166	2.9409	-0.0768	0.0845	0.9989

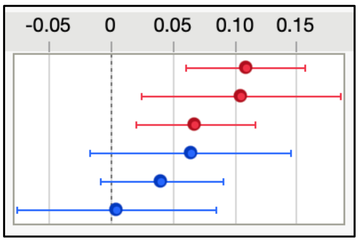

Welch's Test:

F Ratio	DF Num	DF Den	Prob > F
12.8309	3	31.723	<.0001*

Vertebra L2 (L1) Male (Figure 4a): Vertebra body breadth (LL/RL)/ vertebral body length (AP/DV)

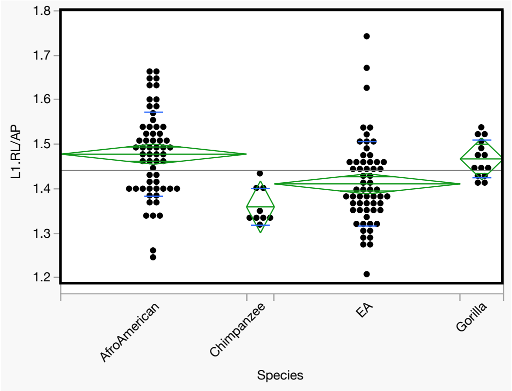

Analysis of Variance:

Source	DF	Sum of Squares	Mean Square	F Ratio	Prob > F
Species	3	0.2052493	0.068416	8.6853	<.0001
Error	139	1.0949449	0.007877		
C. Total	142	1.3001942			

Ordered Differences Report

Species-Male		Difference	Std Err Dif	DF	q*	Lower 95%	Upper 95%	p-Value
AA	Chimp	0.1186	0.0130	23.6713	2.7614	0.0680	0.1693	<.0001*
Gorilla	Chimp	0.1079	0.0126	17.6309	2.8321	0.0575	0.1583	<.0001*
AA	EA	0.0669	0.0122	117.9984	2.6060	0.0218	0.1119	0.0010*
Gorilla	EA	0.0561	0.0118	46.8630	2.6637	0.0116	0.1006	0.0082*
EA	Chimp	0.0518	0.0130	23.7982	2.7603	0.0010	0.1025	0.0443*
AA	Gorilla	0.0107	0.0118	46.6511	2.6641	-0.0337	0.0551	0.9173

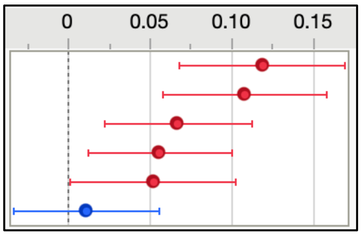

Welch's Test:

F Ratio	DF Num	DF Den	Prob > F
17.5904	3	36.455	<.0001*

Vertebra L3 (L2) Male (Figure 4a): Vertebra body breadth (LL/RL)/ vertebral body length (AP/DV)

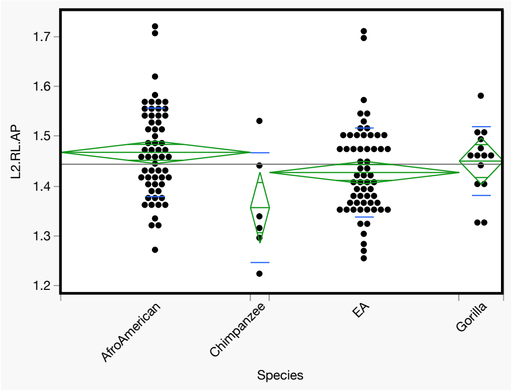

Analysis of Variance:

**Source**	**DF**	**Sum of Squares**	**Mean Square**	**F Ratio**	**Prob > F**
Species	3	0.0969387	0.032313	4.1443	<.0076
Error	136	1.0603790	0.007797		
C. Total	139	1.1573177			

Ordered Differences Report

**Species-Male**		Differ-ence	Std Err Dif	DF	q*	Lower 95%	Upper 95%	p-Value
AA	Chimp	0.111	0.033	5.670	3.526	-0.053	0.275	0.1842
Gorilla	Chimp	0.093	0.034	6.751	3.343	-0.069	0.256	0.3028
EA	Chimp	0.070	0.033	5.678	3.525	-0.093	0.234	0.4856
AA	EA	0.041	0.012	117.997	2.606	-0.002	0.083	0.0668
Gorilla	EA	0.023	0.015	24.334	2.756	-0.037	0.083	0.7188
AA	Gorilla	0.018	0.015	24.209	2.757	-0.042	0.077	0.8505

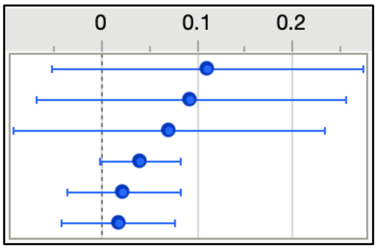

Welch's Test:

F Ratio	DF Num	DF Den	Prob > F
3.1901	3	19.543	<.00465*

Vertebra L4 (L3) Male (Figure 4a): Vertebra body breadth (LL/RL)/vertebral body length (AP/DV).

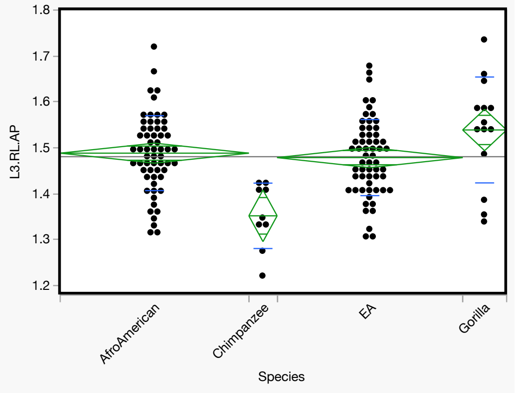

Analysis of Variance:

Source	DF	Sum of Squares	Mean Square	F Ratio	Prob > F
Species	3	0.2009619	0.066987	9.1860	<.0001
Error	138	1.0063438	0.007292		
C. Total	141	1.2073057			

Ordered Differences Report

Species-Male		Difference	Std Err Dif	DF	q*	Lower 95%	Upper 95%	p-Value
Gorilla	Chimp	0.187	0.028	20.993	2.787	0.079	0.296	0.0005*
AA	Chimp	0.137	0.018	11.400	2.992	0.059	0.214	0.0012*
EA	Chimp	0.127	0.018	11.576	2.985	0.049	0.205	0.0021*
Gorilla	EA	0.060	0.023	16.323	2.855	-0.033	0.154	0.2891
Gorilla	AA	0.051	0.023	16.165	2.858	-0.043	0.144	0.4318
AA	EA	0.010	0.011	116.88	2.606	-0.030	0.049	0.9177

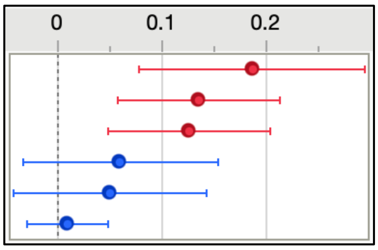

Welch's Test:

F Ratio	DF Num	DF Den	Prob > F
10.4604	3	27.097	<.0001*

Vertebra L5 (L4) Male (Figure 4a): Vertebra body breadth (LL/RL)/ vertebral body length (AP/DV)

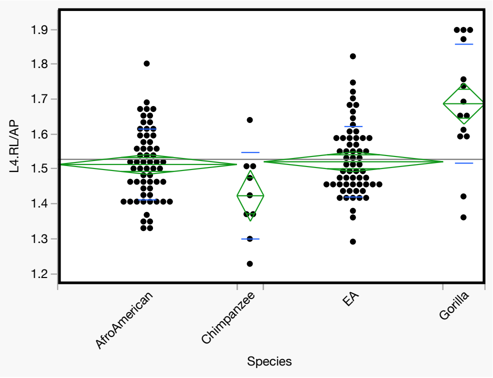

Analysis of Variance:

**Source**	**DF**	**Sum of Squares**	**Mean Square**	**F Ratio**	**Prob > F**
Species	3	0.4692246	0.156408	12.7284	<.0001
Error	139	1.7080479	0.012288		
C. Total	142	2.1772725			

Ordered Differences Report

**Species-Male**		Differ-ence	Std Err Dif	DF	q*	Lower 95%	Upper 95%	p-Value
Gorilla	Chimp	0.264	0.043	20.568	2.792	0.092	0.435	0.0018*
Gorilla	AA	0.174	0.034	15.215	2.877	0.038	0.310	0.0106*
Gorilla	EA	0.166	0.033	15.194	2.878	0.030	0.303	0.0147*
EA	Chimp	0.097	0.031	9.659	3.079	-0.036	0.231	0.1777
AA	Chimp	0.090	0.031	9.675	3.078	-0.044	0.223	0.2298
EA	AA	0.008	0.013	117.99	2.606	-0.040	0.056	0.9750

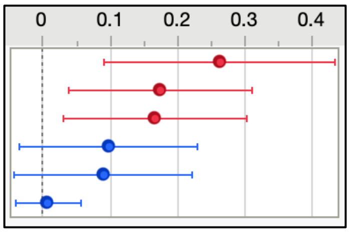

Welch's Test:

F Ratio	DF Num	DF Den	Prob > F
6.0849	3	25.643	<.0029*

Vertebra L1 (T13) Female (Figure 4b): Vertebra body breadth (LL/RL)/ vertebral body length (AP/DV)

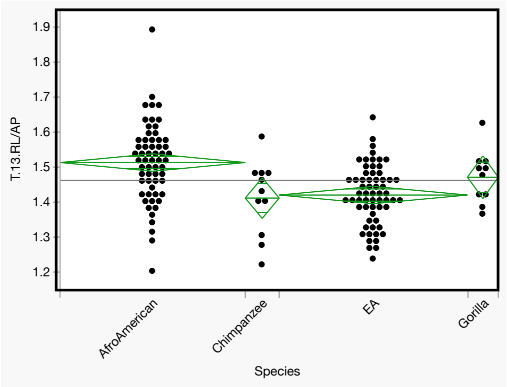

Analysis of Variance:

Source	DF	Sum of Squares	Mean Square	F Ratio	Prob > F
Species	3	0.2923631	0.097454	10.2230	<.0001*
Error	138	1.3155272	0.009533		
C. Total	141	1.6078903			

Ordered Differences Report

Species-Female		Differ-ence	Std Err Dif	DF	q*	Lower 95%	Upper 95%	p-Value
AA	Chimp	0.1016	0.0249	14.2345	2.9005	-0.0008	0.2040	0.0520
AA	EA	0.0929	0.0126	109.966	2.6088	0.0462	0.1397	<.0001*
Gorilla	Chimp	0.0596	0.0284	18.0025	2.8263	-0.0540	0.1733	0.4670
Gorilla	EA	0.0510	0.0185	12.9057	2.9380	-0.0263	0.1282	0.2599
AA	Gorilla	0.0419	0.0197	16.2379	2.8564	-0.0379	0.1218	0.4592
EA	Chimp	0.0086	0.0240	12.3233	2.9573	-0.0919	0.1092	0.9939

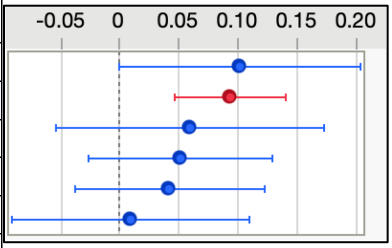

Welch's Test:

F Ratio	DF Num	DF Den	Prob > F
9.2567	3	26.455	0.0002*

Vertebra L2 (L1) Female (Figure 4b): Vertebra body breadth (LL/RL)/ vertebral body length (AP/DV)

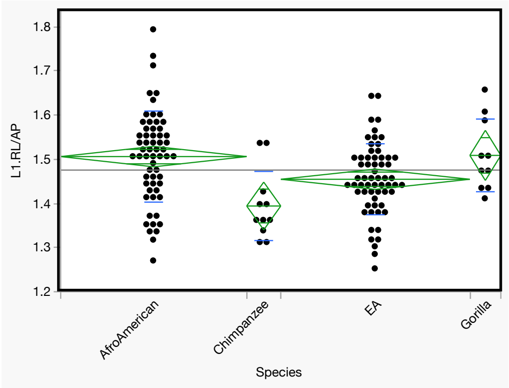

Analysis of Variance:

Source	DF	Sum of Squares	Mean Square	F Ratio	Prob > F
Species	3	0.1661044	0.055368	6.7260	0.0003*
Error	138	1.1360178	0.008232		
C. Total	141	1.3021222			

Ordered Differences Report

Species-Female		Difference	Std Err Dif	DF	q*	Lower 95%	Upper 95%	p-Value
Gorilla	Chimp	0.1145	0.0248	18.579	2.8177	0.0156	0.2135	0.0200*
AA	Chimp	0.1118	0.0192	17.079	2.8412	0.0349	0.1888	0.0035*
EA	Chimp	0.0605	0.0182	14.076	2.9046	-0.0142	0.1353	0.1331
Gorilla	EA	0.0540	0.0198	11.987	2.9693	-0.0290	0.1371	0.2665
AA	EA	0.0513	0.0119	111.44	2.6082	0.0075	0.0952	0.0148*
Gorilla	AA	0.0027	0.0207	14.174	2.9020	-0.0821	0.0875	0.9997

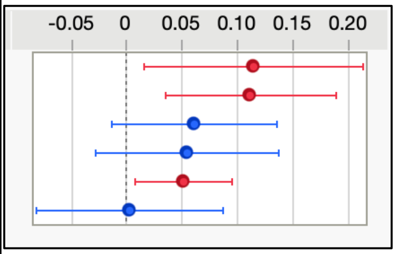

Welch's Test:

F Ratio	DF Num	DF Den	Prob > F
7.0049	3	26.676	0.0013*

Vertebra L3 (L2) Female (Figure 4b): Vertebra body breadth (LL/RL)/ vertebral body length (AP/DV)

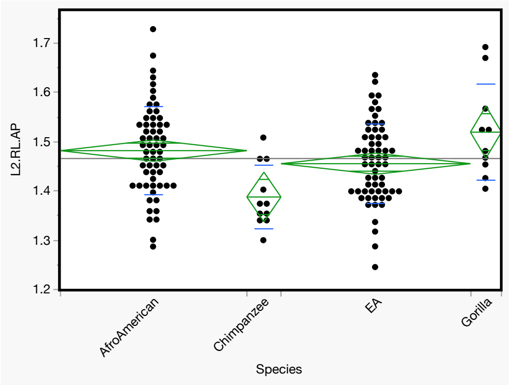

Analysis of Variance L3 (L2) female:

Source	DF	Sum of Squares	Mean Square	F Ratio	Prob > F
Species	3	0.1178972	0.039299	5.4781	0.0014*
Error	138	0.9899894	0.007174		
C. Total	141	1.1078866			

Ordered Differences Report

Species-Female		Difference	Std Err Dif	DF	q*	Lower 95%	Upper 95%	p-Value
Gorilla	Chimp	0.1316	0.0257	15.395	2.8734	0.0268	0.2365	0.0119*
AA	Chimp	0.0941	0.0160	17.906	2.8277	0.0301	0.1581	0.0030*
EA	Chimp	0.0676	0.0155	16.207	2.8569	0.0046	0.1305	0.0330*
Gorilla	EA	0.0640	0.0230	11.108	3.0046	-0.0336	0.1618	0.2560
Gorilla	AA	0.0375	0.0232	11.665	2.9815	-0.0606	0.1358	0.6730
AA	EA	0.0264	0.0109	117.31	2.6062	-0.0139	0.0668	0.3236

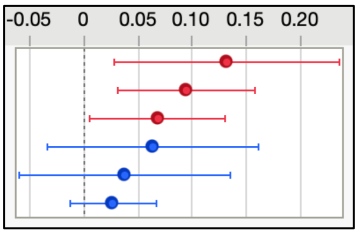

Welch's Test:

F Ratio	DF Num	DF Den	Prob > F
6.7521	3	26.624	0.0016*

Vertebra L4 (L3) Female (Figure 4b): Vertebra body breadth (LL/RL)/ vertebral body length (AP/DV)

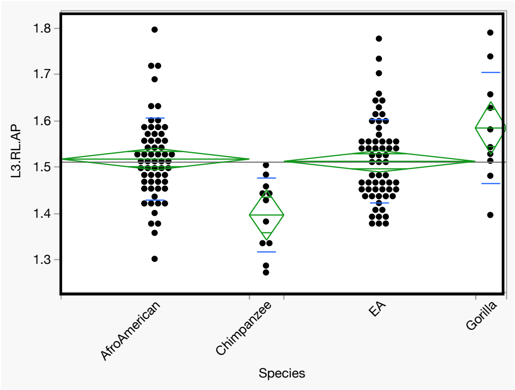

Analysis of Variance:

Source	DF	Sum of Squares	Mean Square	F Ratio	Prob > F
Species	3	0.1999632	0.066654	8.0743	<.0001*
Error	138	1.1392019	0.008255		
C. Total	141	1.3391650			

Ordered Differences Report

**Species-Female**		Difference	Std Err Dif	DF	q*	Lower 95%	Upper 95%	p-Value
Gorilla	Chimp	0.1876	0.0317	15.4442	2.8724	0.0588	0.3165	0.0038*
AA	Chimp	0.1206	0.0188	14.9083	2.8842	0.0439	0.1973	0.0020*
EA	Chimp	0.1158	0.0188	14.9803	2.8826	0.0390	0.1926	0.0029*
Gorilla	EA	0.0718	0.0280	10.7220	3.0223	-0.048	0.1915	0.3190
Gorilla	AA	0.0671	0.0280	10.6983	3.0235	-0.053	0.1867	0.3724
AA	EA	0.0048	0.0115	118.9996	2.6057	-0.037	0.0470	0.9912

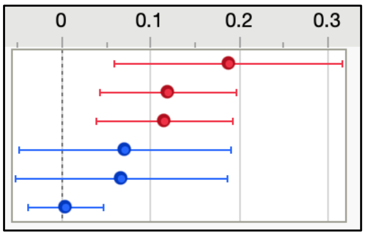

Welch's Test:

F Ratio	DF Num	DF Den	Prob > F
8.3249	3	25.679	0.0005*

Vertebra L5 (L4) Female (Figure 4b): Vertebra body breadth (LL/RL)/ vertebral body length (AP/DV)

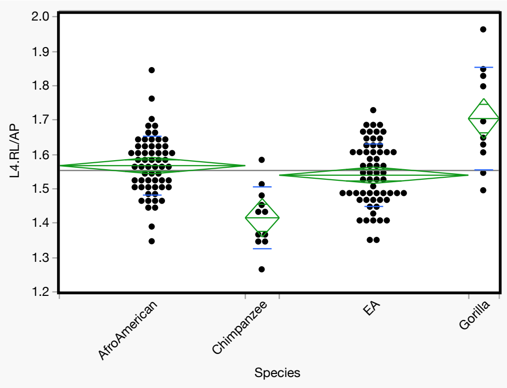

Analysis of Variance:

**Source**	**DF**	**Sum of Squares**	**Mean Square**	**F Ratio**	**Prob > F**
Species	3	0.4594711	0.153157	17.4900	<.0001*
Error	138	1.2084448	0.008757		
C. Total	141	1.6679159			

Ordered Differences Report

**Species-Female**		Differ-ence	Std Err Dif	DF	q*	Lower 95%	Upper 95%	p-Value
Gorilla	Chimp	0.2886	0.0385	14.512	2.8936	0.1312	0.4460	0.0005*
Gorilla	EA	0.1646	0.0343	10.124	3.0526	0.0164	0.3129	0.0291*
AA	Chimp	0.1517	0.0207	13.528	2.9195	0.0662	0.2372	0.0008*
Gorilla	AA	0.1369	0.0342	10.010	3.0588	-0.0113	0.2850	0.0723
EA	Chimp	0.1239	0.0209	13.939	2.9082	0.0381	0.2098	0.0044*
AA	EA	0.0278	0.0113	118.77	2.6058	-0.0141	0.0696	0.3129

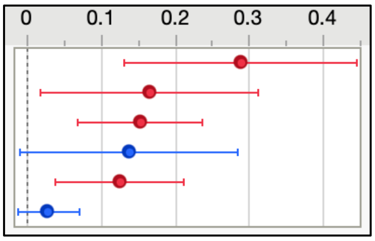

Welch's Test:

F Ratio	DF Num	DF Den	Prob > F
12.1367	3	24.916	<.0001*

Vertebra L1 (T13) Male (Figure 4c): Vertebral body breadth (LL/RL)/ Average vertebral body height (AVBH)

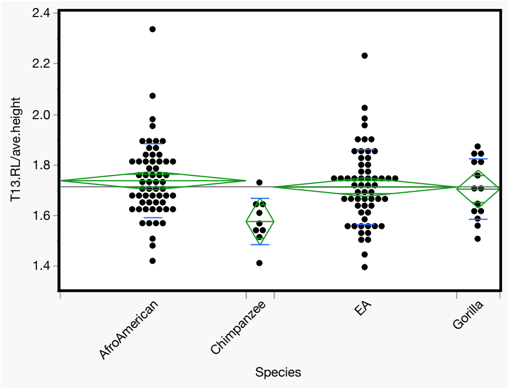

Analysis of Variance:

Source	DF	Sum of Squares	Mean Square	F Ratio	Prob > F
Species	3	0.2051608	0.068387	3.3928	0.0198*
Error	138	2.7816118	0.020157		
C. Total	141	2.9867726			

Ordered Differences Report

Species-Male		Difference	Std Err Dif	DF	q*	Lower 95%	Upper 95%	p-Value
AA	Chimp	0.1612	0.0254	14.9996	2.8822	0.0578	0.2645	0.0022*
EA	Chimp	0.1359	0.0255	15.3198	2.8751	0.0322	0.2396	0.0087*
Gorilla	Chimp	0.1281	0.0312	20.1962	2.7966	0.0047	0.2514	0.0401*
AA	Gorilla	0.0331	0.0262	23.0797	2.7666	-0.0694	0.1356	0.8081
AA	EA	0.0252	0.0191	116.900	2.6064	-0.0450	0.0955	0.7855
EA	Gorilla	0.0079	0.0263	23.5215	2.7627	-0.0950	0.1108	0.9966

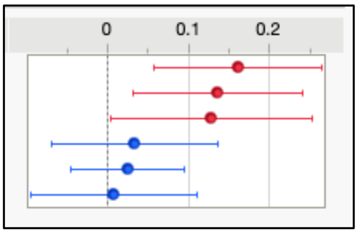

Welch's Test:

F Ratio	DF Num	DF Den	Prob > F
6.6459	3	30.481	0.0014*

Vertebra L2 (L1) male (Figure 4c): Vertebral body breadth (LL/RL)/ Average vertebral body height (AVBH)

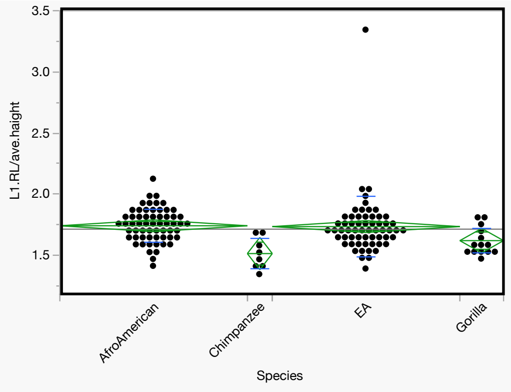

Analysis of Variance:

Source	DF	Sum of Squares	Mean Square	F Ratio	Prob > F
Species	3	0.5144481	0.171483	4.7959	0.0033*
Error	138	4.9343156	0.035756		
C. Total	141	5.4487638			

Ordered Differences Report

Species-Male		Differ-ence	Std Err Dif	DF	q*	Lower 95%	Upper 95%	p-Value
AA	Chimp	0.2273	0.0333	9.35211	3.0980	0.08109	0.37352	0.0039*
EA	Chimp	0.2205	0.0383	15.8930	2.8631	0.06512	0.37601	0.0046*
AA	Gorilla	0.1215	0.0225	25.3337	2.7481	0.03379	0.20929	0.0042*
EA	Gorilla	0.1148	0.0294	52.8943	2.6525	0.00416	0.22544	0.0392*
Gorilla	Chimp	0.1057	0.0363	12.2663	2.9593	-0.04630	0.25783	0.2202
AA	EA	0.0067	0.0257	91.1362	2.6171	-0.08858	0.10205	0.9977

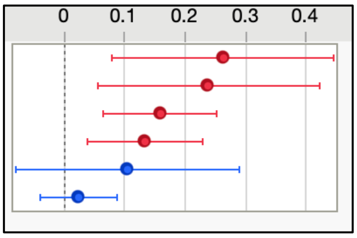

Welch's Test:

F Ratio	DF Num	DF Den	Prob > F
10.675	3	27.511	0.0001*

Vertebra L3 (L2) Male (Figure 4c): Vertebral body breadth (LL/RL)/ Average vertebral body height (AVBH)

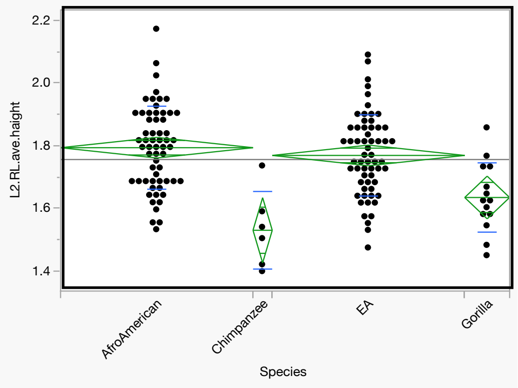

Analysis of Variance:

Source	DF	Sum of Squares	Mean Square	F Ratio	Prob > F
Species	3	0.6059002	0.201967	12.1709	0.0001*
Error	136	2.2568147	0.016594		
C. Total	139	2.8627149			

Ordered Differences Report

Species-Male		Difference	Std Err Dif	DF	q*	Lower 95%	Upper 95%	p-Value
AA	Chimp	0.26324	0.03769	6.2026	3.4262	0.0805	0.44590	0.0095*
EA	Chimp	0.23860	0.03761	6.1491	3.4353	0.0558	0.42134	0.0155*
AA	Gorilla	0.15875	0.02411	22.6082	2.7709	0.0642	0.25324	0.0006*
EA	Gorilla	0.13411	0.02398	22.1725	2.7751	0.0399	0.22823	0.0035*
Gorilla	Chimp	0.10448	0.04136	8.6125	3.1504	-0.0797	0.28876	0.3414
AA	EA	0.02464	0.01689	117.944	2.6060	-0.0376	0.08692	0.7314

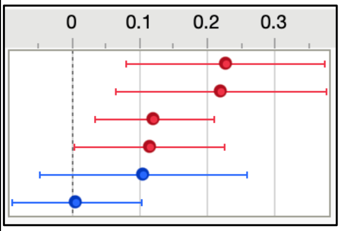

Welch's Test:

F Ratio	DF Num	DF Den	Prob > F
13.1019	3	19.879	0.0001*

Vertebra L4 (L3) Male (Figure 4c): Vertebral body breadth (LL/RL)/ Average vertebral body height (AVBH)

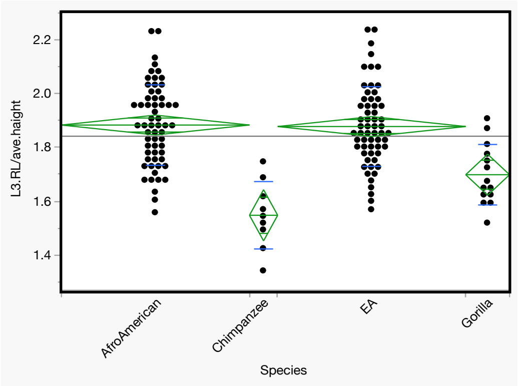

Analysis of Variance:

Source	DF	Sum of Squares	Mean Square	F Ratio	Prob > F
Species	3	1.2459031	0.415301	19.8955	0.0001*
Error	139	2.9015084	0.020874		
C. Total	142	4.1474115			

Ordered Differences Report

Species-Male		Difference	Std Err Dif	DF	q*	Lower 95%	Upper 95%	p-Value
AA	Chimp	0.3355	0.0325	11.7090	2.9799	0.1984	0.4726	<0.0001*
EA	Chimp	0.3299	0.0325	11.6355	2.9827	0.1930	0.4669	<0.0001*
AA	Gorilla	0.1847	0.0253	24.9935	2.7507	0.0865	0.2830	0.0001*
EA	Gorilla	0.1792	0.0252	24.7568	2.7525	0.0811	0.2772	0.0002*
Gorilla	Chimp	0.1508	0.0364	15.8211	2.8646	0.0034	0.2981	0.0440*
AA	EA	0.0055	0.0192	117.9897	2.6060	-0.0652	0.0763	0.9970

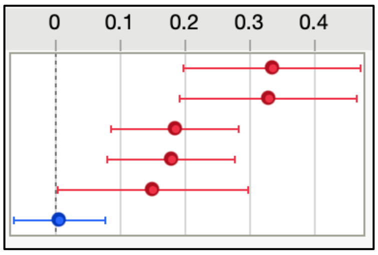

Welch's Test:

F Ratio	DF Num	DF Den	Prob > F
25.0127	3	28.835	0.0001*

Vertebra L5 (L4) Male (Figure 4c): Vertebral body breadth (LL/RL)/ Average vertebral body height (AVBH)

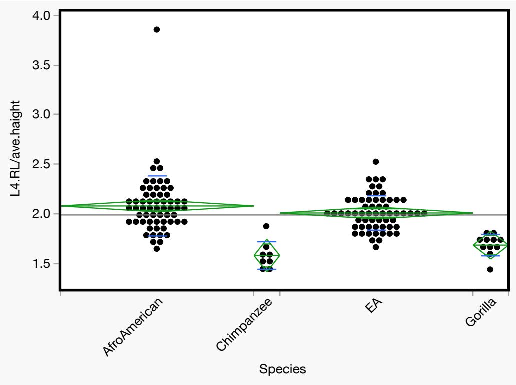

Analysis of Variance:

Source	DF	Sum of Squares	Mean Square	F Ratio	Prob > F
Species	3	2.860980	0.953660	17.3719	0.0001*
Error	135	7.411063	0.054897		
C. Total	138	10.272043			

Ordered Differences Report

Species-Male		Differ-ence	Std Err Dif	DF	q*	Lower 95%	Upper 95%	p-Value
AA	Chimp	0.49866	0.0442	17.9098	2.8277	0.3217	0.6756	<.0001*
EA	Chimp	0.42793	0.0380	10.1667	3.0503	0.2640	0.5919	<.0001*
AA	Gorilla	0.39401	0.0360	43.7198	2.6707	0.2580	0.5301	<.0001*
EA	Gorilla	0.32328	0.0280	20.7011	2.7907	0.2128	0.4338	<.0001*
Gorilla	Chimp	0.10464	0.0416	12.8683	2.9392	-0.0683	0.2776	0.3265
AA	EA	0.07072	0.0318	93.8221	2.6157	-0.0469	0.1884	0.3991

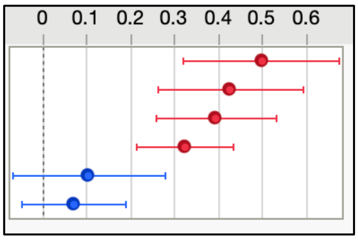

Welch's Test:

F Ratio	DF Num	DF Den	Prob > F
41.3970	3	26.363	0.0001*

Figure 4d-Female

Vertebra L1 (T13) Female (Figure 4d): Vertebral body breadth (LL/RL)/ Average vertebral body height (AVBH)

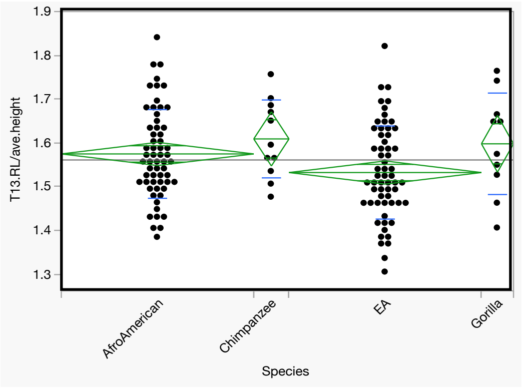

Analysis of Variance:

**Source**	**DF**	**Sum of Squares**	**Mean Square**	**F Ratio**	**Prob > F**
Species	3	0.1000747	0.033358	3.0988	0.0289*
Error	137	1.4747907	0.010765		
C. Total	140	1.57486			

Ordered Differences Report

Species-Female		Difference	Std Err Dif	DF	q*	Lower 95%	Upper 95%	p-Value
Chimp	AA	0.0343	0.0211	15.175	2.8782	-0.0517	0.1203	0.6664
Gorilla	AA	0.0231	0.0275	11.412	2.9918	-0.0933	0.1396	0.9318
Chimp	EA	0.0768	0.0213	15.738	2.8663	-0.0096	0.1633	0.0904
Gorilla	EA	0.0657	0.0277	11.667	2.9815	-0.0511	0.1824	0.3772
AA	EA	0.0425	0.0134	117.73	2.6061	-0.0069	0.0920	0.1181
Chimp	Gorilla	0.0112	0.0321	16.870	2.8448	-0.1181	0.1405	0.9946

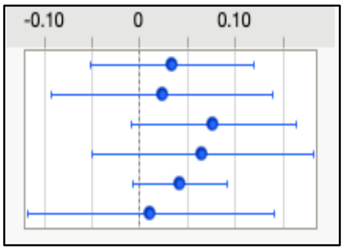

Welch's Test:

F Ratio	DF Num	DF Den	Prob > F
3.0695	3	26.232	0.0453*

Vertebra L2 (L1) Female (Figure 4d): Vertebral body breadth (LL/RL)/ Average vertebral body height (AVBH)

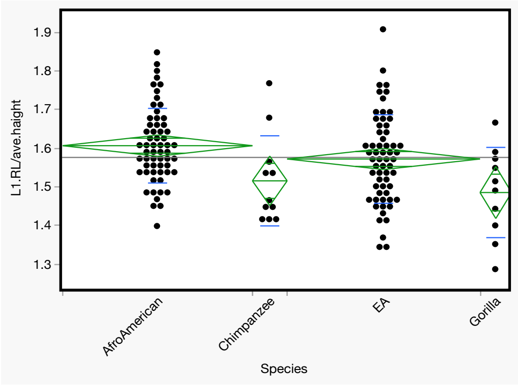

Analysis of Variance:

Source	DF	Sum of Squares	Mean Square	F Ratio	Prob > F
Species	3	0.1790827	0.059694	5.1670	0.0020*
Error	138	1.5943073	0.011553		
C. Total	141	1.7733900			

Ordered Differences Report

Species-Female		Difference	Std Err Dif	DF	q*	Lower 95%	Upper 95%	p-Value
AA	Gorilla	0.1211	0.0276	11.1444	3.0031	0.00412	0.2381	0.0419
AA	Chimp	0.0909	0.0264	12.6371	2.9467	-0.01894	0.2008	0.1196
EA	Gorilla	0.0867	0.0281	12.0163	2.9682	-0.03120	0.2046	0.1832
EA	Chimp	0.0565	0.0269	13.7121	2.9143	-0.05447	0.1675	0.472
AA	EA	0.0344	0.0136	116.286	2.6066	-0.01571	0.0846	0.283
Chimp	Gorilla	0.0302	0.0360	18.8046	2.8145	-0.11326	0.1737	0.933

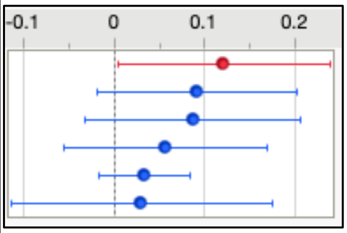

Welch's Test:

F Ratio	DF Num	DF Den	Prob > F
4.6677	3	25.392	0.0099*

Vertebra L3 (L2) Female (Figure 4d): Vertebral body breadth (LL/RL)/ Average vertebral body height (AVBH)

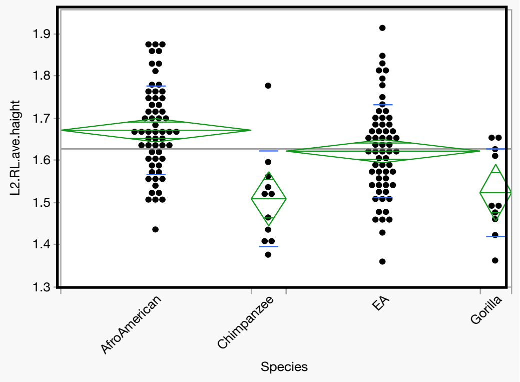

Analysis of Variance:

Source	DF	Sum of Squares	Mean Square	F Ratio	Prob > F
Species	3	0.380	0.127	10.952	0.0001*
Error	138	1.597	0.0115		
C. Total	141	1.977			

Ordered Differences Report

Species-Female		Difference	Std Err Dif	DF	q*	Lower 95%	Upper 95%	p-Value
AA	Chimp	0.16	0.03	13.3	2.93	0.05	0.27	0.00
AA	Gorilla	0.15	0.03	12.3	2.96	0.04	0.25	0.01
EA	Chimp	0.11	0.03	13.6	2.92	0.01	0.22	0.04
EA	Gorilla	0.10	0.03	12.6	2.95	-0.0	0.20	0.07
AA	EA	0.05	0.01	119	2.61	-0.0	0.10	0.06
Gorilla	Chimp	0.01	0.03	19.0	2.81	-0.1	0.15	0.99

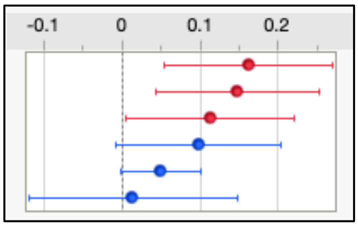

Welch's Test:

F Ratio	DF Num	DF Den	Prob > F
10.3957	3	25.971	0.0001*

Vertebra L4 (L3) Female (Figure 4d): Vertebral body breadth (LL/RL)/ Average vertebral body height (AVBH)

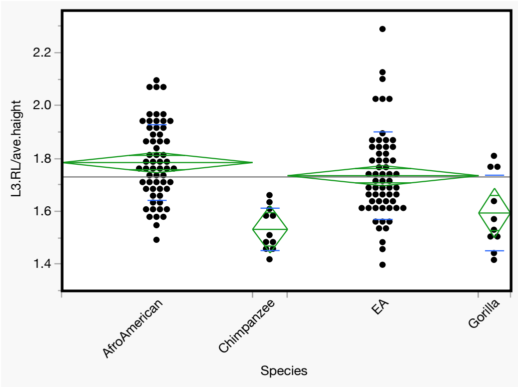

Analysis of Variance:

Source	DF	Sum of Squares	Mean Square	F Ratio	Pro>F
Species	3	0.7966686	0.265556	11.8416	<.0001
Error	137	3.0723247	0.022426		
C. Total	140	3.8689934			

Ordered Differences Report

Species-Female		Differ-ence	Std Err Dif	DF	q*	Lower 95%	Upper 95%	p-Value
AA	Chimp	0.2526	0.021	23.737	2.761	0.169	0.336	<.0001
EA	Chimp	0.2029	0.023	28.819	2.726	0.115	0.291	<.0001
AA	Gorilla	0.1907	0.035	12.204	2.962	0.046	0.335	0.0095
EA	Gorilla	0.1411	0.035	13.377	2.924	-0.005	0.287	0.0600
Gorilla	Chimp	0.0618	0.036	13.857	2.910	-0.087	0.211	0.6327
AA	EA	0.0496	0.020	115.554	2.607	-0.024	0.123	0.2994

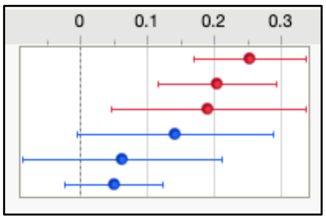

Welch's Test:

F Ratio	DF Num	DF Den	Prob > F
24.72	3	29.439	0.0001*

Vertebra L5 (L4) Female (Figure 4d): Vertebral body breadth (LL/RL)/ Average vertebral body height (AVBH)

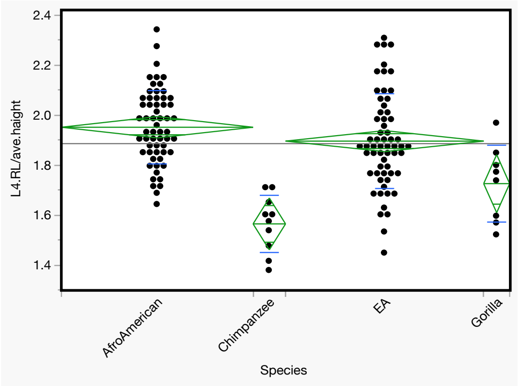

Analysis of Variance:

Source	DF	Sum of Squares	Mean Square	F Ratio	Pro>F
Species	3	1.5001012	0.500034	18.1175	<.0001
Error	134	3.6983239	0.027599		
C. Total	137	5.1984251			

Ordered Differences Report

Species-Female		Differ-ence	Std Err Dif	DF	q*	Lower 95%	Upper 95%	p-Value
AA	Chimp	0.3872	0.0290	14.5444	2.8928	0.2687	0.5057	<.0001
EA	Chimp	0.3316	0.0308	18.3820	2.8206	0.2086	0.4547	<.0001
AA	Gorilla	0.2260	0.0408	8.8189	3.1348	0.0451	0.4068	0.0158
EA	Gorilla	0.1704	0.0421	10.0258	3.0579	-0.0119	0.3527	0.0686
Gorilla	Chimp	0.1612	0.0462	12.6425	2.9465	-0.0314	0.3538	0.1141
AA	EA	0.0556	0.0219	112.6166	2.6078	-0.0251	0.1362	0.2804

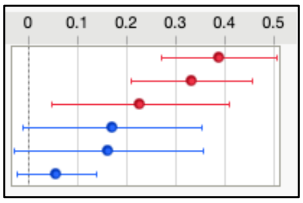

Welch's Test:

F Ratio	DF Num	DF Den	Prob > F
30.7187	3	22.632	0.0001*

Supplemantary Materials Section S2

Statistical analyses for Figure 12: Anterior (**a**) and posterior (**b**) apophyseal ring width relative to vertebral body length (AP/DV), lateral right apophyseal ring width relative to vertebral body breadth (LL/RL), (**c**), and total ring area relative to vertebral body superior discal surface area (**d**) in humans, gorillas, and chimpanzees. Data for males only (ordered difference report and Welch's Test).

Anterior ring width/vertebral body length (AP/DV) (Figure 12a)

Vertebra	Humans *vs*. Gorilla	Humans *vs*. Chimpanzee	Gorilla *vs*. Chimpanzee
T4	0.0001*	0.0674	0.5227
T5	0.0001*	0.0227	0.6545
T6	0.0001*	<.0001*	0.8844
T7	<.0001*	0.0040*	0.5805
T8	<.0001*	0.0108*	0.6168
T9	<.0001*	0.0297*	0.3668
T10	<.0001*	0.0273*	0.0156*
T11	<.0001*	0.0216*	0.1400
T12	<.0001*	0.0026*	0.5172
L1 (T13)	<.0001*	0.0011*	0.0032*
L2 (L1)	<.0001*	0.0013*	0.1543
L3 (L2)	<.0001*	0.0011*	0.1939
L4 (L3)	<.0001*	0.0091*	0.3463
L5 (L4)	<.0001*	0.0003*	0.0090*

Welche's Test:

Verte-bra	F Ratio	DFNum	DFDen	P
T4	20.9433	2	13.252	<.0001*
T5	34.8986	2	14.54	<.0001*
T6	45.5195	2	14.195	<.0001*
T7	37.1176	2	12.126	<.0001*
T8	69.1928	2	12.023	<.0001*
T9	35.8948	2	13.48	<.0001*
T10	37.3318	2	13.612	<.0001*
T11	51.0609	2	12.444	<.0001*
T12	60.9271	2	13.334	<.0001*
L1 (T13)	86.8198	2	13.771	<.0001*
L2 (L1)	64.8473	2	13.513	<.0001*
L3 (L2)	66.7036	2	9.9014	<.0001*
L4 (L3)	44.0530	2	13.4	<.0001*
L5 (L4)	48.5562	2	14.736	<.0001*

Posterior ring width/vertebral body length (AP/DV) (Figure 12b)

Vertebra Number	Humans *vs.* Gorilla	Humans *vs.* Chimpanzee	Gorilla *vs.* Chimpanzee
T4	0.0219*	0.0026*	0.9148
T5	0.045*	<0.0001*	0.9044
T6	0.0018*	<.0001*	0.6835
T7	0.0022*	0.0201*	0.8881
T8	0.0008*	0.0004*	.04622
T9	0.0045*	0.0177*	0.6864
T10	0.0028*	0.0253*	0.9767
T11	0.0028*	0.1881	0.3116
T12	0.0006*	0.0969	0.7616
L1 (T13)	0.0012*	0.0066*	0.4016
L2 (L1)	0.0001*	0.0115*	0.5931
L3 (L2)	0.0001*	0.1556	0.4882
L4 (L3)	0.0013*	0.0533	0.2862
L5 (L4)	<.0001*	0.0032*	0.1761

Welch's Test:

Vertebra	F Ratio	DFNum	DFDen	P
T4	12.7105	2	15.274	<.0006*
T5	27.8646	2	16.434	<.0001*
T6	34.6141	2	16.722	<.0001*
T7	14.5181	2	11.721	0.0007*
T8	25.7601	2	14.71	<.0001*
T9	12.910	2	13.183	0.0008*
T10	12.5668	2	13.459	0.0008*
T11	11.0849	2	12.019	0.0019*
T12	14.5049	2	11.326	0.0008*
L1 (T13)	14.0941	2	11.681	0.0008*
L2 (L1)	26.3122	2	9.0467	0.0002*
L3 (L2)	21.4869	2	8.5283	0.0005*
L4 (L3)	13.227	2	12.078	0.00089*
L5 (L4)	28.1799	2	13.52	<.0001*

Lateral (right) apophyseal ring width/vertebral body breadth (LL/RL) (Figure 12 c)

Vertebra Number	Humans *vs.* Gorilla	Humans *vs.* Chimpanzee	Gorilla *vs.* Chimpanzee
T4	<.0001*	0.0050*	0.9985
T5	0.0011*	<.0001*	0.5506
T6	<.0001*	0.0002*	0.9571
T7	0.0004*	<.0001*	0.9964
T8	<.0001*	0.0126*	0.7382
T9	<.0001*	<.0030*	0.3321
T10	<.0001*	0.0016*	0.344*
T11	<.0001*	0.0142*	0.2286
T12	<.0001*	<.0046*	0.4089
L1 (T13)	<.0001*	0.0115*	0.4448
L2 (L1)	<.0001*	0.0737	0.0548
L3 (L2)	<.0001*	0.0780	0.2109
L4 (L3)	0.0006*	0.0925	0.0682
L5 (L4)	0.0063*	0.1748	0.4829

Welch's Test:

Verte-bra	F Ratio	DFNum	DFDen	P
T4	30.1262	2	12.308	<.0001*
T5	42.4751	2	13.099	<.0001*
T6	53.3357	2	14.545	<.0001*
T7	44.6347	2	14.064	<.0001*
T8	48.0703	2	12.534	<.0001*
T9	43.0427	2	13.717	<.0001*
T10	55.7702	2	13.837	<.0001*
T11	43.9365	2	12.547	<.0001*
T12	39.2734	2	13.691	<.0001*
L1(T13)	36.8120	2	13.805	<.0001*
L2 (L1)	33.5357	2	13.821	<.0001*
L3 (L2)	22.7293	2	10.178	0.0002*
L4 (L3)	14.5306	2	14.2	0.0004*
L5 (L4)	8.1503	2	14.197	0.0044*

Total ring area/discal surface area (Figure 12d)

Vertebra Number	Humans *vs.* Gorilla	Humans *vs.* Chimpanzee	Gorilla *vs.* Chimpanzee
T7	0.0007*	0.0001*	0.6891
T8	0.0039*	0.0001*	0.9762
T9	0.0007*	<.0001*	0.8989
T10	0.0020*	0.0003*	0.9960
T11	<.0001*	0.0068*	0.7709
T12	<.0001*	0.0094*	0.9061
L1 (T13)	0.0002*	0.0031*	0.8835
L2 (L1)	<.0001*	0.0056*	0.8201
L3 (L2)	0.0004*	0.00286*	0.5585
L4 (L3)	0.0013*	0.1921	0.2258
L5 (L4)	0.0090*	0.2337	0.2797

Welch's Test:

Vertebra	F Ratio	DFNum	DFDen	P
T7	29.8029	2	14,829	<.0001*
T8	25.1613	2	14.007	<.0001*
T9	34.1684	2	14.434	<.0001*
T10	25.1656	2	14.044	<.0001*
T11	28.4157	2	14.026	<.0001*
T12	26.4077	2	14.083	<.0001*
L1 (T13)	23.8103	2	14.474	<.0001*
L2 (L1)	27.0847	2	14.54	<.0001*
L3 (L2)	17.0102	2	10.842	<.0001*
L4 (L3)	10.6860	2	14.803	0.0013*
L5 (L4)	6.5667	2	16.349	0.0085*

Multiple regression analyses (Log transformed data):

Dependent variable – discal surface area

Independent variables – age, sex, body height, body weight, waist circumference at the level of L3-L4, and cortical shell thickness

Summary of fit

Column 1	Column 2
RSquare	0.479651
RSquare Adj	0.343908
Root Mean Square Error	0.050676
Mean of Response	3.115534

Analysis of variance

Source	DF	Sum of Squares	Mean Square	F Ratio	Prob > F
Model	6	0.054445	0.009074	3.533518	0.012592*
Error	23	0.059065	0.002568		
C. Total	29	0.113511			

Parameter estimates

Term	Estimate	Std Error	t Ratio	Prob>|t|
Intercept	2.767317	0.372679	7.425473	<.0001*
age	0.000925	0.000955	0.9686	0.3428
Log WCIRL3	-0.06237	0.351737	-0.17731	0.8608
Log Height	1.512434	0.705725	2.143091	0.0429*
Log Weight	0.056425	0.224266	0.251601	0.8036
Log mean cortical thickness	-0.06784	0.220569	-0.30759	0.7612
Male-Female	-0.00856	0.014679	-0.58329	0.5654

Effect test

Source	Nparm	DF	Sum of Squares	F Ratio	Mean Square	Prob > F
age	1	1	0.002409	0.938187	0.002409	0.3428
Log WCIRL3	1	1	8.07E-05	0.031438	8.07E-05	0.8608
Log Height	1	1	0.011795	4.592838	0.011795	0.0429*
Log Weight	1	1	0.000163	0.063303	0.000163	0.8035
Log mean cortical thickness	1	1	0.000243	0.094612	0.000243	0.7612
Male-Female	1	1	0.000874	0.340232	0.000874	0.5654
